# Cysteine desulfurase (IscS)–mediated fine-tuning of bioenergetics and SUF expression prevents *Mycobacterium tuberculosis* hypervirulence

**DOI:** 10.1126/sciadv.adh2858

**Published:** 2023-12-13

**Authors:** Mayashree Das, Sreesa Sreedharan, Somnath Shee, Nitish Malhotra, Meghna Nandy, Ushashi Banerjee, Sakshi Kohli, Raju S. Rajmani, Nagasuma Chandra, Aswin Sai Narain Seshasayee, Sunil Laxman, Amit Singh

**Affiliations:** ^1^ Department of Microbiology and Cell Biology, Indian Institute of Science, Bangalore 560012, India.; ^2^ Centre for Infectious Disease Research, Indian Institute of Science, Bangalore 560012, India.; ^3^ Institute for Stem Cell Science and Regenerative Medicine, Bangalore 560065, India.; ^4^ School of Chemical and Biotechnology, (SASTRA)-Deemed to be University, Thanjavur 613401, India.; ^5^ National Centre for Biological Sciences (NCBS), Tata Institute of Fundamental Research (TIFR), Bangalore 560065, India.; ^6^ Department of Biochemistry, Indian Institute of Science, Bangalore 560012, India.; ^7^ Molecular Biophysics Unit, Indian Institute of Science, Bangalore 560012, India.

## Abstract

Iron-sulfur (Fe-S) biogenesis requires multiprotein assembly systems, SUF and ISC, in most prokaryotes. *M. tuberculosis* (*Mtb*) encodes a complete SUF system, the depletion of which was bactericidal. The ISC operon is truncated to a single gene *iscS* (cysteine desulfurase), whose function remains uncertain. Here, we show that *Mtb*Δ*iscS* is bioenergetically deficient and hypersensitive to oxidative stress, antibiotics, and hypoxia. *Mtb*Δ*iscS* resisted killing by nitric oxide (NO). RNA sequencing indicates that IscS is important for expressing regulons of DosR and Fe-S–containing transcription factors, WhiB3 and SufR. Unlike wild-type *Mtb*, *Mtb*Δ*iscS* could not enter a stable persistent state, continued replicating in mice, and showed hypervirulence. The *suf* operon was overexpressed in *Mtb*Δ*iscS* during infection in a NO-dependent manner. Suppressing *suf* expression in *Mtb*Δ*iscS* either by CRISPR interference or upon infection in inducible NO-deficient mice arrests hypervirulence. Together, *Mtb* redesigned the ISC system to “fine-tune” the expression of SUF machinery for establishing persistence without causing detrimental disease in the host.

## Teaser

Coordinated regulation of IscS and Suf-mediated Fe-S cluster homeostasis controls persistence of Mycobacterium tuberculosis.

## INTRODUCTION

The *Mycobacterium tuberculosis* (*Mtb*) life cycle inside the human host relies upon successful adaptation to commonly encountered stresses such as reactive oxygen species (ROS), reactive nitrogen species (RNS), iron starvation, low pH, and hypoxia (*1*). Iron-sulfur (Fe-S) clusters, which are the most ancient protein prosthetic groups, are sensitive targets for ROS and RNS, and their biogenesis is adversely affected by iron starvation (*2*, *3*). *Mtb* contains more than 50 Fe-S cluster proteins that carry out diverse functions within central metabolism, gene regulation, drug resistance, and persistence (table S1) (*4*, *5*). Therefore, knowledge of the biogenesis and repair of Fe-S clusters is critical to understanding the basis of persistence for this human pathogen.

Many organisms express multiple Fe-S assembly systems (e.g., NIF, ISC, and SUF), wherein the SUF system is largely restricted to organisms that are frequently exposed to Fe-S cluster–damaging conditions (*6*). In line with this, *Mtb* expresses a complete SUF system (*Rv1461-Rv1466*) that not only is essential under standard growth conditions but also protects *Mtb* from stresses [e.g., nitric oxide (NO) and iron starvation] by repairing damaged Fe-S clusters (*7*–*9*). Furthermore, depletion of the SUF system impairs *Mtb’s* ability to maintain redox balance, central carbon metabolism (CCM), respiration, and persistence when in animals (*10*, *11*). Consistent with these findings, the SUF system was induced under stressful conditions such as ROS, reactive nitrogen intermediate (RNI), low iron, antibiotics, macrophage milieu, and sputum of tuberculosis (TB) patients (*9*, *12*–*16*). *Mtb* also encodes an *iscS* gene (*Rv3025c*; cysteine desulfurase) that is not a part of the *suf* locus and is not surrounded by other *isc* genes, such as *iscA* and *iscU*, and by chaperones *hscA* and *hscB* (*17*). Although these findings indicate that the SUF machinery is the primary Fe-S biogenesis system in *Mtb*, a previous report suggested the involvement of *Mtb* IscS in building a 4Fe-4S cluster of the transcription factor WhiB3 in vitro (*18*). Moreover, a mutant of IscS (*Mtb*Δ*iscS*) was hypersensitive to oxidative stress and displayed impaired activity of the Fe-S cluster–dependent enzymes, aconitase and succinate dehydrogenase (*17*). These findings indicate that IscS is involved in maintaining Fe-S cluster homeostasis and protects *Mtb* from oxidative stress. However, important questions remain unanswered: What are the mechanisms by which IscS contributes to oxidative stress resistance and what is the consequence of an IscS loss in the backdrop of a fully functional SUF system on the persistence and virulence of *Mtb*.

In this study, we used a redox biosensor and extracellular flux (XF) analyzer to compare the redox balance and bioenergetics of wild-type (WT) *Mtb* and *Mtb*Δ*iscS*. We also examined metabolomics and transcriptomics of *Mtb*Δ*iscS* and analyzed the survival phenotype of the mutant under diverse stresses in vitro and in mice. Last, we found that the interplay between IscS and SUF system is crucial for adjusting the virulence of *Mtb* in mice. We anticipate that understanding the IscS-linked metabolic and regulatory events will contribute toward knowledge of how *Mtb* maintain cellular homeostasis for persistence.

## RESULTS

### IscS is required to maintain redox homeostasis in *Mtb*

**Fig. 1.**
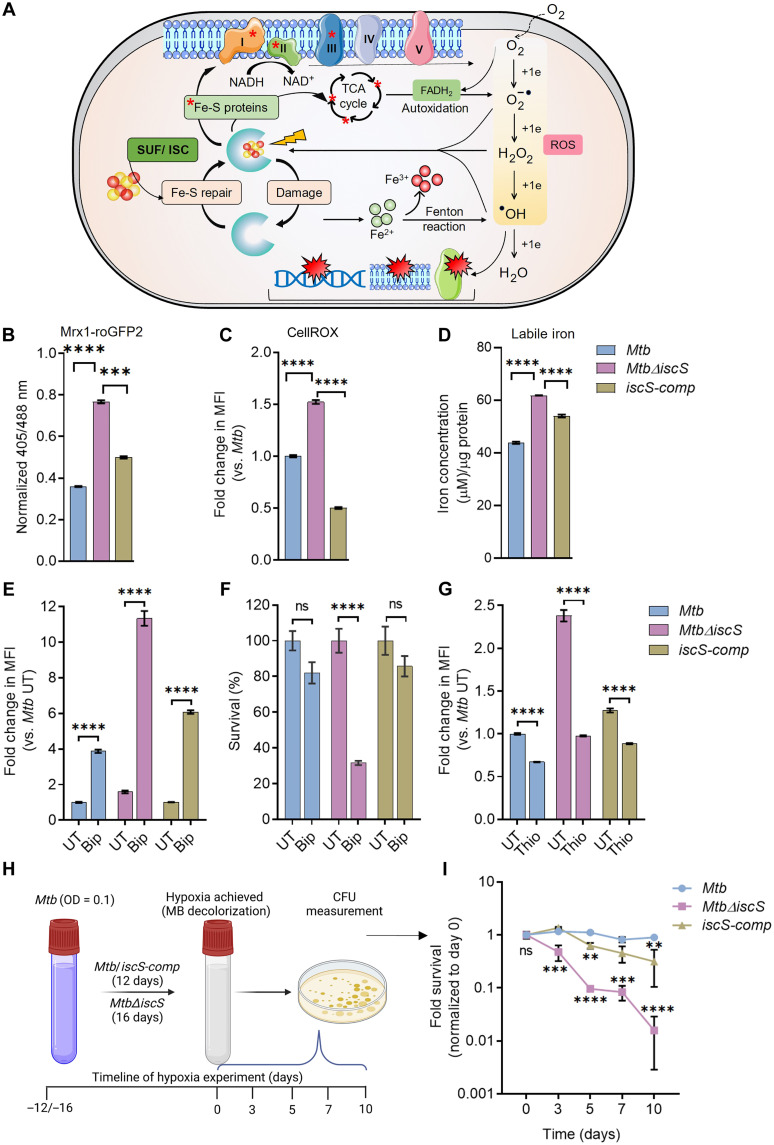
IscS is required to maintain redox balance of *Mycobacterium tuberculosis *(*Mtb*). (**A**) Diagrammatic representation of endogenous reactive oxygen species (ROS) generation (O_2_^−•^, H_2_O_2_, and ^•^OH) due to the univalent reduction of O_2_ via electron leak from redox-active enzymes. O_2_^−•^ and H_2_O_2_ disrupt iron-sulfur (Fe-S) clusters, resulting in leaching of iron and formation of highly deleterious ^•^OH radical by Fenton reaction. SUF and ISC pathways are essential for Fe-S cluster biogenesis/repair. (**B**) The mycothiol redox potential (*E*_MSH_) of *Mtb*, *Mtb*Δ*iscS*, *and iscS-comp* was determined by measuring Mrx1-roGFP2 biosensor ratio (405/488 nm) using flow cytometry. (**C**) *Mtb*, *Mtb*Δ*iscS*, *and iscS-comp* were stained with CellROX Deep Red dye to measure endogenous ROS. (**D**) Intracellular labile iron was determined for *Mtb*, *Mtb*Δ*iscS*, and *iscS-comp* by ferrozine-based colorimetric assay. Iron concentration was normalized to the protein content. *Mtb*, *Mtb*Δ*iscS*, *and iscS-comp* were exposed to 250 μM of cell-permeable iron chelator 2,2-bipyridyl (Bip) for 24 hours, followed by measurement of (**E**) endogenous ROS and (**F**) survival. (**G**) Endogenous ROS of *Mtb*, *Mtb*Δ*iscS*, and *iscS-comp* upon treatment with ROS scavenger Thio (10 mM). (**H**) Schematic representation showing experimental strategy to measure persistence of *Mtb* under hypoxia (credit: BioRender.com). (**I**) Viability of *Mtb*, *Mtb*Δ*iscS*, *and iscS-comp* cultured under hypoxia by colony-forming unit (CFU) enumeration. Data are presented as means ± SEM. (B to D) *P ≤ 0.05 and ****P ≤ 0.0001 by one-way analysis of variance (ANOVA) with Bonferroni’s multiple comparisons test. (E to G and I) **P ≤ 0.01, ***P ≤ 0.001, and ****P ≤ 0.0001; ns, not significant by two-way ANOVA with Bonferroni’s multiple comparisons test.

A previous study reported that depletion of IscS results in a slightly slower growth rate of *Mtb* and diminished activity of Fe-S cluster–containing enzyme, aconitase, under aerobic culture conditions (*17*). We confirmed this observation using *Mtb*Δ*iscS* (fig. S1, A and B). Aerobic metabolism inevitably generates highly deleterious superoxide (O_2_^-•^) and hydrogen peroxide (H_2_O_2_) due to the univalent reduction of molecular oxygen (O_2_) by redox enzymes (Fig. 1A). ROS-scavenging enzymes, along with Fe-S cluster biogenesis systems, protect cells from the adverse consequences of oxidative radicals and maintains redox homeostasis in diverse organisms (*2*, *5*, *19*–*21*). Therefore, we asked whether IscS is required to maintain redox balance under aerobic growth conditions in *Mtb*. To do this, we used a genetically encoded redox biosensor, Mrx1-roGFP2, to measure the redox potential of the antioxidant buffer mycothiol [reduced mycothiol (MSH)/oxidized mycothione (MSSM); *E*_MSH_] as a proxy for cytoplasmic redox state of *Mtb* (*22*). The readout of Mrx1-roGFP2 can be analyzed by measuring the fluorescence intensity at 510 nm emission and excitation at 405 and 488 nm (fig. S2). An increase in the 405/488 ratio indicates a shift in the MSH/MSSM ratio toward MSSM due to either enzymatic oxidation of MSH in response to ROS or depletion of the total mycothiol pool (*22*). Treatment of *Mtb*-expressing Mrx1-roGFP2 to increasing concentrations of H_2_O_2_ gradually increased the biosensor ratio (fig. S3).

Exponentially growing *Mtb*Δ*iscS* expressing the biosensor exhibited a ~1.5- to 2.0-fold higher 405/488 ratio than WT *Mtb* and *iscS-com*p (Fig. 1B), indicating that the mutant suffers from oxidative stress under aerobic conditions. We have previously reported that a twofold increase in the biosensor ratio is similar to the oxidative stress induced by 500 μM H_2_O_2_ (fig. S3). These changes in the biosensor ratio corresponds to slightly oxidized *E*_MSH_ of −260 mV for *Mtb*Δ*iscS* as compared to WT *Mtb* (*E*_MSH_ = −280 mV) and *iscS*-*comp* (−275 mV). Using CellROX Deep Red dye, which becomes fluorescent upon intracellular oxidation by ROS, we confirmed that *Mtb*Δ*iscS* stained with CellROX displayed 1.5- and 3.0-fold greater fluorescence than WT *Mtb* and *iscS-comp*, respectively (Fig. 1C). An equivalent increase in CellROX fluorescence was observed upon treatment of *Mtb* with 50 to 100 μM cumene hydroperoxide (CHP) (fig. S4). ROS are known to damage Fe-S clusters and increase the pool of labile iron (*19*). We confirmed this by showing higher ROS in *Mtb* growing under iron-excess conditions, whereas iron limitation decreases ROS levels (fig. S5). Consistent with this finding, we observed an ~1.5-fold increase in free iron in *Mtb*Δ*iscS* compared to WT *Mtb* (Fig. 1D). Collectively, these data suggest that aerobically grown *Mtb*Δ*iscS* displays disruption of redox and iron homeostatic mechanisms.

Because most of the cytoplasmic pool of labile iron exists in the ferrous form (*23*) and catalyzes the Fenton reaction to generate deleterious hydroxyl radicals, we investigated whether ROS accumulation and slow growth of *Mtb*Δ*iscS* are due to elevated iron in the mutant. We treated exponentially grown WT *Mtb*, *Mtb*Δ*iscS*, and *iscS-comp* with 250 μM of the cell-permeable iron chelator 2,2-bipyridyl (Bip) followed by measurement of ROS and viability at 24 hours after treatment. Iron deprivation by Bip induces the IdeR-dependent expression of *esx-3* and *suf* operons (*13*). We detected increased expression of *esx-3* (*esxH*) and *suf* genes (*sufR*, *sufS*, and *sufB*) in Bip-treated *Mtb*, confirming iron deprivation (fig. S1C). Surprisingly, CellROX staining revealed higher ROS in all three strains upon treatment with Bip (Fig. 1E). While Bip treatment increased ROS in WT *Mtb* (~4-fold) and *iscS-comp* (~6-fold), a >10-fold increase was observed in *Mtb*Δ*iscS* as compared to WT *Mtb* untreated (Fig. 1E). Consistent with this, Bip treatment reduced the viability of *Mtb*Δ*iscS* by 70%. In contrast, Bip treatment reduced viability by only 10 to 20% in WT *Mtb* and *iscS-comp* (Fig. 1F). We determined the minimal inhibitory concentration at 90% (MIC_90_) of Bip for WT *Mtb*, *Mtb*ΔiscS, and *iscS-comp* using Microplate Alamar Blue Assay (MABA). A twofold lower MIC_90_ of Bip was observed in the case of *Mtb*Δ*iscS* compared to WT *Mtb* and *iscS-comp* (fig. S1D). While surprising, our findings correlate with work on obligate aerobes and photosynthetic organisms such as *Anabaena* sp. and *Caulobacter cresentus* that display heightened ROS under iron starvation—a phenomenon not detected in facultative anaerobes/aerobes, such as *Escherichia coli* and *Bacillus subtilis* (*24*, *25*).

Given that a deficiency of IscS leads to accumulation of ROS in *Mtb*, treatment with antioxidants could potentially rescue the slow growth phenotype of *Mtb*Δ*iscS*. To test this, we evaluated the effect of thiourea (Thio), an ROS scavenger (*20*), on ROS accumulation using CellROX and survival of *Mtb*Δ*iscS.* As expected, treatment with 10 mM Thio for 24 hours reduced ROS levels in *Mtb*Δ*iscS* (Fig. 1G). All three strains showed comparable levels of ROS upon Thio treatment (Fig. 1G). Surprisingly, despite lowering ROS levels, Thio treatment further inhibited the growth of *Mtb*Δ*iscS* as revealed by two- to fourfold lower MIC_90_ of Thio against *Mtb*Δ*iscS* (3.125 mM) than WT *Mtb* (6.25 mM) and *iscS-comp* (12.5 mM) (fig. S1D).

Intracellular ROS generation takes place when flavoenzymes inadvertently relocate a fraction of their electron flux directly to molecular oxygen (*26*). Thus, under oxygen-deficient conditions, we expect a reduction in ROS generation. We hypothesize that if ROS accumulation is the cause of slow growth of *Mtb*Δ*iscS*, we could rescue the growth phenotype of mutant under conditions that limit ROS formation such as oxygen depletion. We used an in vitro Wayne model of hypoxia and reaeration (*27*). In this model, *Mtb* respiration gradually uses oxygen such that hypoxia and bacteriostasis is achieved by day 12 (Fig. 1H). We examined the growth phenotype of WT *Mtb*, *Mtb*Δ*iscS*, and *iscS-comp* after establishment of hypoxia in the Wayne model (*27*). We monitored oxygen depletion by examining decoloration of the dye methylene blue (MB). As expected, the blue color of MB dye decolorized by day 12 for WT *Mtb* and *iscS-comp*. However, and consistent with the slow growth phenotype of the mutant, the color of MB dye faded by day 16 for *Mtb*Δ*iscS*. We followed the viability of three strains for 10 days after establishment of hypoxia (i.e., after dye decolorization). As previously reported (*28*), WT *Mtb* retained 100% viability under hypoxic conditions for the entire duration of the experiment (Fig. 1I). In contrast, *Mtb*Δ*iscS* showed gradual loss of viability over time under hypoxic conditions (Fig. 1I). We conclude that increased ROS accumulation during aerobic metabolism might not be the primary cause of slow growth of *Mtb*Δ*iscS*. Because IscS coordinates Fe-S clusters of metabolic (e.g., aconitase) and respiratory (e.g., succinate dehydrogenase) enzymes (*17*), impaired bioenergetics could lead to ROS accumulation and slow growth of *Mtb*Δ*iscS*. Aberrant respiration could trap components of the electron transport chain in the reduced state that can directly transfer electron to molecular oxygen leading to ROS formation (*29*, *30*). These events are possible as enough molecular oxygen might be present even under hypoxia for the reduction and ROS formation (*31*, *32*).

### IscS deficiency affects CCM in *Mtb*

**Fig. 2.**
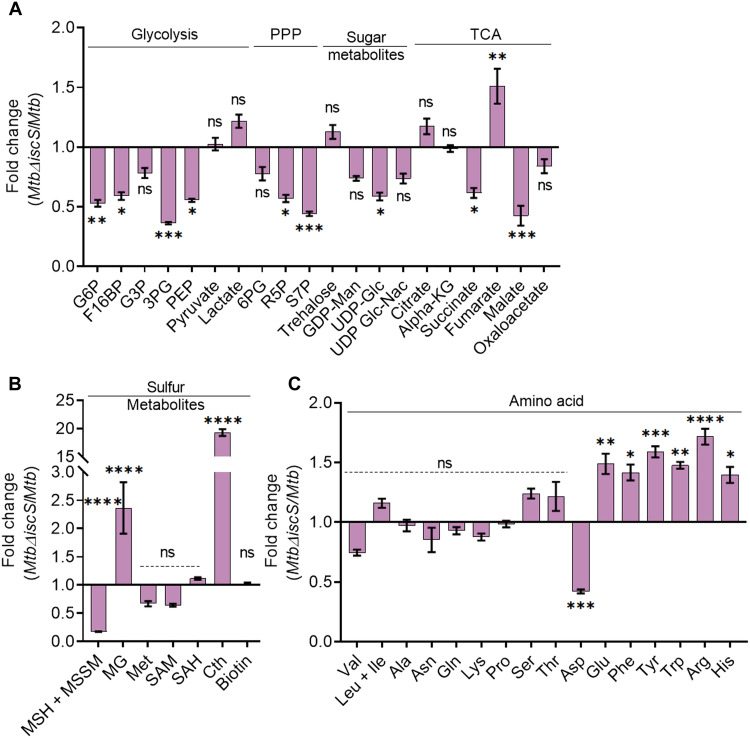
IscS deletion results in deregulation of central carbon metabolism in *Mycobacterium tuberculosis *(*Mtb*). Quantitative liquid chromatography–mass spectrometry (LC-MS/MS) analysis of (**A**) glycolytic intermediates, pentose phosphate metabolites, tricarboxylic acid (TCA) metabolites, sugar nucleotides, (**B**) sulfur metabolites, and (**C**) amino acids of *Mtb* and *Mtb*Δ*iscS*. Data are presented as fold change respective to *Mtb* and means ± SEM. *P ≤ 0.05, **P ≤ 0.01, ***P ≤ 0.001, and ****P ≤ 0.0001 by two-way analysis of variance (ANOVA) with Bonferroni’s multiple comparisons test compared to *Mtb* levels. PPP, pentose phosphate pathway; G6P, glucose-6-phosphate/fructose-6-phosphate; F16BP, fructose-1,6-bisphosphate; G3P, glyceraldehyde-3-phosphate; 3PG, 3-phosphoglycerate; PEP, phosphoenolpyruvate; MG, methylglyoxal; Met, methionine; SAM, *S*-adenosyl methionine; SAH, *S*-adenosyl homocysteine; Cth, cystathionine; 6PG, 6-phosphogluconate; R5P, ribulose-5-phosphate; S7P, sedoheptulose-7-phosphate; GDP-Man, guanosine diphosphate mannose; UDP-Glc, uracil diphosphate glucose; UDP-Glc-Nac, uridine diphosphate *N*-acetylglucosamine; ns, not significant.

Fe-S cluster–dependent enzymes play key roles in CCM, branched-chain amino acid synthesis, and nucleotide biosynthesis (*33*). To examine whether the growth defect exhibited by *Mtb*Δ*iscS* correlates with a consequence of impaired metabolism, we used targeted, quantitative liquid chromatography–mass spectrometry (LC-MS/MS). We quantified the steady-state amounts of glycolytic, pentose phosphate pathway (PPP), and tricarboxylic acid (TCA) intermediates. We uniformly observed decreased amounts of several glycolytic and PPP intermediates in *Mtb*Δ*iscS* as compared to WT *Mtb* (Fig. 2A). The influence of IscS deficiency on glycolysis and PPP was surprising, as none of the glycolytic and PPP enzymes contains Fe-S clusters. However, several glycolytic and gluconeogenesis enzymes contain cysteine thiols, which are sensitive to inhibition by oxidation and S-mycothiolation in mycobacteria (*34*, *35*). Further, glucose-6-phosphate (G6P) levels are regulated by endogenous ROS (*36*); therefore, diminished G6P levels in the mutant could be a consequence of ROS accumulation in *Mtb*Δ*iscS*. Efficient metabolism of G6P is also essential for generating nicotinamide adenine dinucleotide phosphate hydrogen (NADPH) through PPP (*36*), which serves as reductive power for antioxidant buffers (e.g., mycothiol and thioredoxin systems) and cell wall lipid biogenesis. Consistent with diminished PPP intermediates, levels of NADPH were slightly reduced, and nicotinamide adenine dinucleotide phosphate (NADP^+^) levels were increased in *Mtb*Δ*iscS*, resulting in an overall increase in NADP^+^/NADPH ratio as compared to WT *Mtb* (fig. S6, A to C).

**Fig. 3.**
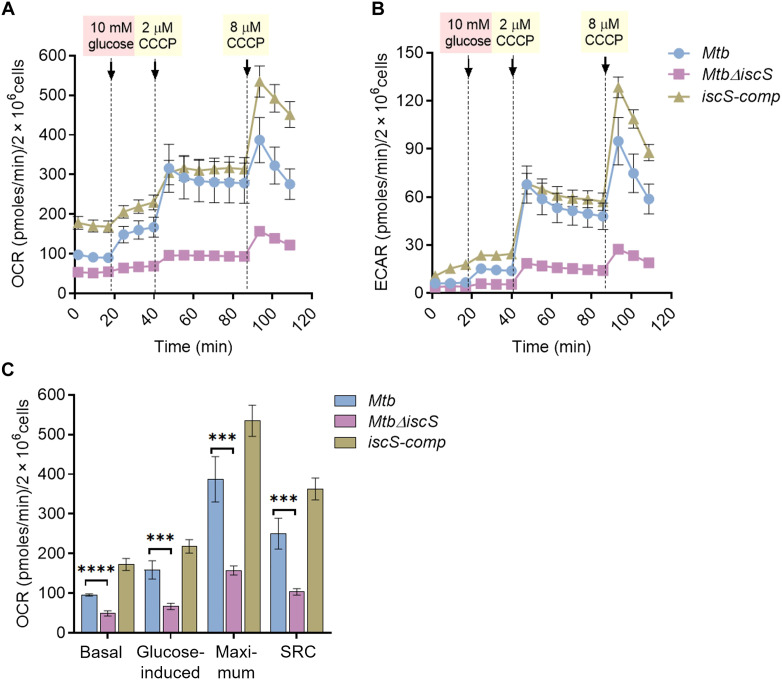
Deletion of IscS results in diminished oxygen consumption rate (OCR) and extracellular acidification rate (ECAR). (**A**) OCR and (**B**) ECAR measurement of *Mycobacterium tuberculosis * (*Mtb*) , *Mtb*Δ*iscS*, and *iscS-comp* after injection of glucose and the uncoupler carbonyl cyanide *m*-chlorophenyl hydrazine (CCCP) indicated by the dotted lines. (**C**) Graph plotting basal respiration, glucose-induced respiration, maximum respiratory, and spare respiratory capacity (SRC) as derived from OCR values. All points of OCR and ECAR are normalized to colony-forming unit (CFU) (2 × 10^6^ cells per well). Data are presented as means ± SEM. *P ≤ 0.05, **P ≤ 0.01, ***P ≤ 0.001, and ****P ≤ 0.0001 calculated by unpaired two-tailed *t* test.

Impaired glycolysis and PPP are also reflected in a lower abundance of sugar metabolites uridine 5′-diphosphate glucose (UDP-Glc) along with a modest reduction in guanosine diphosphate mannose (GDP-MAN) and uridine diphosphate *N*-acetyl glucosamine (UDP-Glc-NAC) required for the biosynthesis of peptidoglycan (Fig. 2A) (*37*). UDP-Glc-NAC is an essential substrate for mycothiol glycosyltransferase, which catalyzes the first step in the biosynthesis of mycothiol in *Mtb* (*38*). As expected, the total mycothiol (MSH + MSSM) content was substantially diminished in *Mtb*Δ*iscS* as compared to WT *Mtb* (Fig. 2B*; P* ≤ 0.001), correlating well with the ratiometric increase in the mycothiol-specific biosensor (Mrx1-roGFP2) observed in the mutant. Mycothiol is also essential for detoxifying a highly reactive dicarbonyl by-product, methylglyoxal (MG) (*38*). We found that MG levels accumulated in *Mtb*Δ*iscS* as compared to WT *Mtb*, and this could in part be linked to the lack of mycothiol-linked remediation (Fig. 2B).

*Mtb*Δ*iscS* has been previously reported to display diminished activities of the Fe-S cluster–containing TCA cycle enzymes aconitase and succinate dehydrogenase (*17*). Inactivation of aconitase is expected to raise citrate levels and reduce downstream TCA cycle metabolites such as α-ketoglutarate (α-KG) (*11*). Citrate showed only a marginal increase. Likewise, α-KG was unaffected in *Mtb*Δ*iscS* (Fig. 2A). Lowered succinate dehydrogenase activity indicates that the conversion of succinate to fumarate would be deficient in *Mtb*Δ*iscS*. Surprisingly, rather than accumulating succinate, *Mtb*Δ*iscS* displayed succinate depletion along with a concomitant increase in fumarate as compared to WT *Mtb* (Fig. 2A). These results suggest impairment of activity or expression of fumarate reductase in *Mtb*Δ*iscS.* Fumarate reductase is a 4Fe-4S cluster enzyme encoded by *frdABCD* and catalyzes the conversion of succinate to fumarate (*39*). Along with fumarate accumulation, a notable depletion of malate was observed in *Mtb*Δ*iscS* (Fig. 2A), indicating weakened fumarate hydratase activity. In line with the defective TCA cycle, the generation of nicotinamide adenine dinucleotide hydrogen (NADH) was marginally reduced, leading to higher nicotinamide adenine dinucleotide (NAD^+^) and NAD^+^/NADH ratio in *Mtb*Δ*iscS* as compared to WT *Mtb* and *IscS-comp* (fig. S6, D to F). A massive accumulation of the transsulfuration pathway metabolite cystathionine (Cth; 20-fold) and minor albeit nonsignificant decrease in methionine (Met) and *S*-adenosyl methionine (SAM) suggests an impairment of sulfur metabolism in *Mtb*Δ*iscS* (Fig. 2B). The enzyme adenosine 5′-phosphosulfate reductase (CysH) that catalyzes reductive assimilation of inorganic sulfate into Met, SAM, and cysteine (Cys) in *Mtb* contains a 4Fe-4S cluster (*5*); the activity of this enzyme may also be insufficient in *Mtb*Δ*iscS*. Under such conditions (abnormal reduction of inorganic sulfate), *Mtb* relies upon reverse transsulfuration (RTS) enzymes Cth-β-synthase (CBS) that converts homocysteine to Cth and Cth-γ-lyase (MetB) that generates Cys for mycothiol biosynthesis from Cth (*40*, *41*). MetB is also a bifunctional enzyme that can catalyze a γ-replacement reaction using Cys and *O*-succinyl/acetyl-homoserine to generate Cth via forward transsulfuration (FTS) reaction for regenerating methyl cycle intermediates [Met, SAM, and *S*-adenosyl homocysteine (SAH)]. While *Mtb*Δ*iscS* accumulates Cth and maintains methyl cycle intermediates, it shows depletion of mycothiol. These findings suggest that in the absence of IscS, *Mtb* switches from synthesizing Cys via the RTS pathway to using Cys for generating methyl cycle intermediates via the formation of Cth intermediate through the FTS pathway. We recently showed that metabolic switching from RTS to FTS pathways ensured *Mtb* survival by avoiding excess oxidative stress (*40*). Future experiments are required to clarify the link between IscS, RTS, and FTS pathways.

We observed a notable accumulation of various amino acids (Phe, Tyr, Trp, Glu, Arg, and His) in the mutant (Fig. 2C), suggesting attempts of *Mtb* to balance the loss of TCA cycle intermediates by anaplerosis in the absence of IscS. For example, elevated Glu could support the replenishment of α-KG by glutamate dehydrogenase in the background of defective glycolysis and aconitase activity in *Mtb*Δ*iscS*. Despite the reduction in levels of 2-phosphoenol pyruvate (PEP) and PPP intermediates, which are the initial substrates for the biosynthesis of aromatic amino acids (shikimate pathway), *Mtb*Δ*iscS* showed an accumulation of Phe, Trp, and Tyr. This could come from an impairment in the catabolic pathways for these amino acids, for instance, utilization into TCA or cofactor generation (e.g., molybdopterin biosynthesis) (*42*), along with increased utilization of PPP intermediates for amino acid synthesis. Complementation of IscS expression readjusted abundance of ~70% of the metabolites to levels comparable to those detected in WT *Mtb* (fig. S7, A and B). Last, the highlighted changes in the abundance of glycolysis, PPP, TCA, and amino acid pathways metabolites are also observed in the WhiB3 mutant of *Mtb* (*43*). IscS directly interacts with WhiB3 to coordinate 4Fe-4S cluster in vitro, indicating that IscS can regulate the Fe-S cluster–dependent regulatory activity of WhiB3 (*18*). Overall, the data indicate an important interplay between IscS, CCM, sulfur metabolism, and redox homeostasis in *Mtb*.

### IscS is required to maintain bioenergetic homeostasis of *Mtb*

The altered pool of CCM in *Mtb*Δ*iscS* and the dependency of mycobacterial respiration on Fe-S cluster homeostasis (*10*, *11*) motivated us to measure the bioenergetics of the mutant. We used the XF analyzer and noninvasively tracked the extracellular acidification rate (ECAR) and oxygen consumption rate (OCR) of *Mtb*Δ*iscS*. ECAR is an accurate indication of proton (H^+^) translocation due to glycolytic and TCA cycle activity, whereas OCR is a measure of respiration linked to oxidative phosphorylation.

We starved *Mtb* cultures overnight, followed by seeding in the XF microchamber, exposed them to glucose at a specific time, and then treated cells twice with the uncoupler carbonyl cyanide *m*-chlorophenyl hydrazine (CCCP). The injection of CCCP stimulates maximal respiration by *Mtb*, which provides an estimate of the bioenergetic reserves of the cells (*44*). The data are normalized to an equal number of viable cells (2 × 10^6^ per well) in each case. We noticed that the basal OCR (before the addition of glucose) of *Mtb*Δ*iscS* is twofold (*P* = 0.0001) reduced as compared to WT *Mtb* (Fig. 3, A and C) with a change of 1.6-fold (*P* = 0.005) in ECAR (Fig. 3B). In the presence of glucose, *Mtb*Δ*iscS* displayed a substantial reduction in OCR (~2.5-fold, *P* = 0.008) and ECAR (~2.6-fold, *P =* 0.00007) relative to WT *Mtb* (Fig. 3, A to C). As expected, two sequential additions of CCCP stimulated OCR and ECAR in WT *Mtb* (Fig. 3, A and B). In contrast, a negligible increase in OCR and ECAR was observed in the case of CCCP-treated *Mtb*Δ*iscS* (Fig. 3, A and B). As a result, *Mtb*Δ*iscS* showed a 2.4-fold reduction in the spare respiratory capacity as compared to WT *Mtb* (Fig. 3C). The IscS complemented strain largely restored the bioenergetic profile to that of WT *Mtb* (Fig. 3, A to C). We conclude that IscS is required for maintaining the optimal bioenergetics of *Mtb*.

### IscS modulates the expression of DosR, WhiB3, and SufR regulons in *Mtb*

We next asked whether any transcriptional response was associated with the redox, metabolic, and bioenergetic changes we described in *Mtb*Δ*iscS*. We analyzed the global transcriptome of *Mtb*Δ*iscS* compared to WT *Mtb*. Total bacterial RNA was isolated from logarithmically growing cultures at an absorbance of ~0.4, sequenced, and analyzed using the EdgeR platform. Multidimensional scaling analysis showed that the samples clustered by their biological replicates (fig. S8A). Compared to the transcriptome of WT *Mtb*, the expression of 547 genes was altered in *Mtb*Δ*iscS* [false discovery rate (FDR) ≤ 0.05, absolute fold change ≥ 1.5], of which 181 genes were up-regulated, whereas 366 genes showed down-regulation (table S2).

**Fig. 4.**
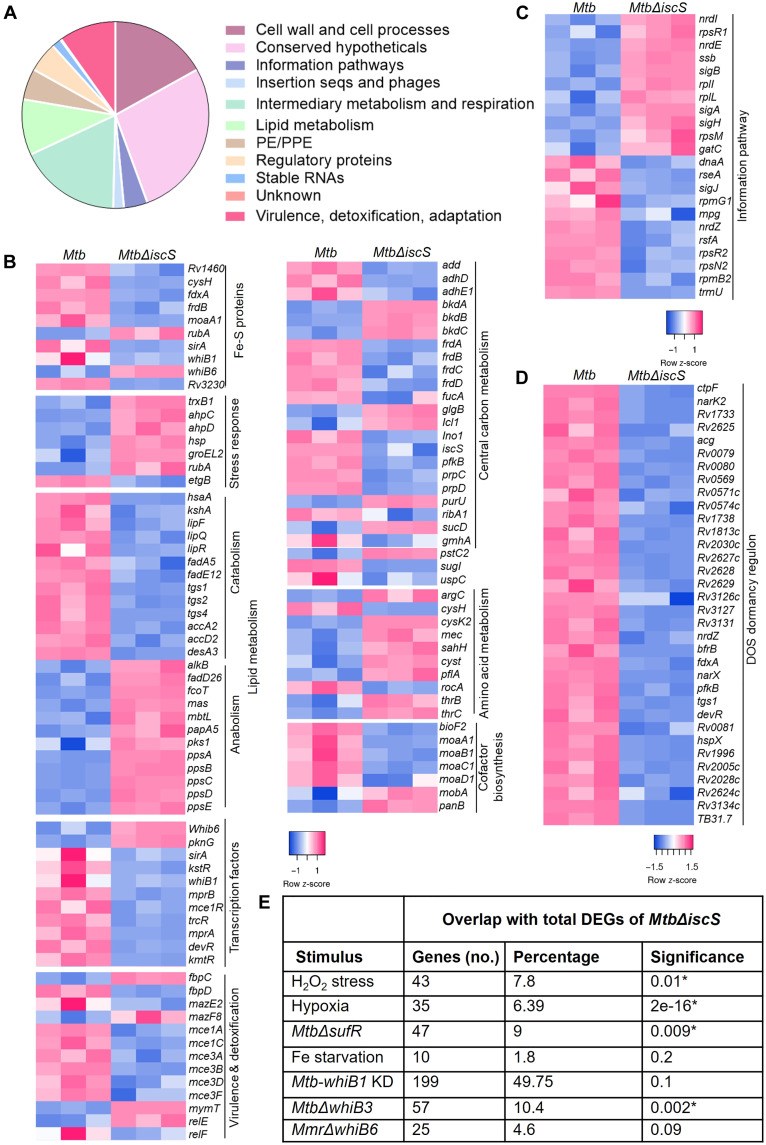
*Mtb*Δ*iscS* displayed altered expression of genes regulated by iron-sulfur (Fe-S)–containing WhiBs, SufR, and DosR/S/T system. Total RNA was isolated from *Mycobacterium tuberculosis * (*Mtb*) cultures, grown to 0.4 OD_600_, and subjected to RNA sequencing (RNA-seq) analysis. (**A**) Mycobrowser-based classification of the genes/pathways [1.5-fold change; false discovery rate (FDR) ≤ 0.05] deregulated in *Mtb*Δ*iscS*. (**B** to **D**) Heatmaps showing gene expression changes belonging to various functional categories. Heatmaps were constructed with row z-score on normalized logCPM values. (**E**) The table summarizes the overlap between the IscS transcriptome with the whole-genome expression under different stress conditions and transcriptomes *of Mtb-whiB1 KD*, *Mtb*Δ*whiB3*, and *Mtb*Δ*sufR*. Fisher’s exact test with *P < 0.05 as a cutoff for significance (table S2).

Classification of differentially expressed genes (DEGs) according to annotated functional categories revealed up-regulation of stress-responsive genes (*trxB1*, *ahpCD*, *hsp*, *rubA*, and *groEL2*) that corroborates our finding of increased ROS in *Mtb*Δ*iscS* (Fig. 4, A and B). Genes involved in lipid metabolism showed a distinct expression pattern, with many catabolic genes (*kshA*, *lipFQR*, *fadA5*, *tgs1*,*2*,*4*, *accA2*, *accD2*, and *desA3*) being repressed; anabolic genes (*alkB*, *fadD*, *mas*, *pks1*, and *ppsA-E*) and lipid transporters (*mce1* and *mce3* operons) were up-regulated. Several genes involved in virulence and detoxification, such as toxin counterparts of toxin-antitoxin systems (*mazF8* and *relE*) (Fig. 4B) and stress-responsive sigma factors (*sigA*, *sigB*, and *sigH*) (Fig. 4C), were induced in *Mtb*Δ*iscS*. In agreement with our metabolomics data, genes associated with CCM such as fumarate reductase (*frdA-D*), phosphofructokinase (*pfkB*), methyl citrate cycle (*prpCD*), inositol-1-phosphate synthase (*ino1*), and molybdenum and biotin biosynthesis (*moaA1-D1* and *bioF2*) were repressed, whereas amino acid metabolism was induced (Fig. 4B). A general shutdown of housekeeping pathways was also evident by the down-regulation of genes involved in DNA and ribosomal RNA (rRNA) synthesis (Fig. 4C).

As expected from a defective adaptation of *Mtb*Δ*iscS* to O_2_ limitation, the hypoxia-responsive Dos-dormancy regulon was uniformly suppressed in the mutant (Fig. 4D). Furthermore, we compared the expression of the Dos-dormancy regulon under hypoxic conditions by performing quantitative reverse transcription polymerase chain reaction (qRT-PCR) of a set of genes (*hspX*, *fdxA*, *devR*, *devS*, and *narK2*) representing the Dos regulon. As expected, hypoxia induces the expression of Dos regulon genes in WT *Mtb*. However, the expression of Dos genes is either less induced (*hspX* and *fdxA*) or down-regulated (*devR*, *devS*, and *nark2*) in *Mtb*Δ*iscS* compared to WT *Mtb* or *iscS-comp* (fig. S9A). We also found that the expression of eight genes encoding Fe-S cluster proteins was repressed, while two genes (*rubA* and *whiB6*) were induced in *Mtb*Δ*iscS* as compared to WT *Mtb* (Fig. 4B). We reasoned that the observed transcriptional changes are not a direct consequence of *iscS* loss but rather due to defective occupancy of Fe-S clusters on transcription factors. Consistent with this idea, the expression of several Fe-S cluster–containing transcription factors, such as *whiB1*, *whiB6*, *sirA*, and *sufR*, was deregulated in *Mtb*Δ*iscS* (Fig. 4B). Moreover, the transcriptome of *Mtb*Δ*iscS* overlapped with that of previously reported regulons of Fe-S cluster–containing transcription factors: *sufR* and *whiB3* (Fig. 4E) (*10*, *45*). Because *whiB1* is essential and its transcriptome is not reported, we depleted *whiB1* in *Mtb* (*whiB1-KD*), using tetracycline-based CRISPR interference (CRISPRi) (fig. S10, A and B), and performed RNA sequencing (RNA-seq). An ~50% overlap between the transcriptomes of *whiB1* and *iscS* was observed (Fig. 4E). We also compared the overlap between DEGs in *Mtb*Δ*iscS* with DEGs of *Mtb* grown under oxygen limitation, H_2_O_2_, and Fe^−^ starvation. We considered that DEGs overlapped irrespective of whether the genes are similarly regulated (induced or repressed) between *Mtb*Δ*iscS* and WT *Mtb* exposed to different stress conditions. On this basis, the RNA-seq data of *Mtb*Δ*iscS* overlapped with *Mtb* grown under oxygen limitation and H_2_O_2_. Similarly, the transcriptome of *Mtb*Δ*iscS* did not coincide with iron starvation (*13*), a finding consistent with an increased fraction of free iron in the *iscS* mutant (Fig. 4E). The *iscS-comp* strain notably complements the IscS deregulated transcripts (table S2).

### IscS protects *Mtb* from lethality caused by anti-TB drugs

**Fig. 5.**
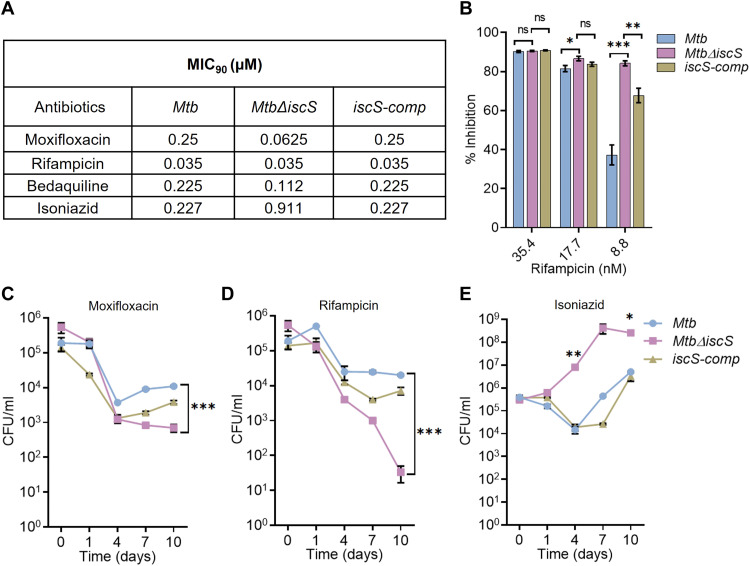
*Mtb*Δ*iscS* shows enhanced sensitivity to anti-tuberculosis (TB) drugs. (**A**) *Mtb*, *Mtb*Δ*iscS*, and *iscS-comp* grown until OD_600_ values of 0.6 were treated with various concentrations of Rif, Mox, Bdq, and Inh, and MIC_90_ was determined using Alamar blue assay. Data are representative of two independent experiments done in duplicate. (**B**) Alamar blue dataset was used to calculate percent inhibition of *Mtb*Δ*iscS* by Rif compared to *Mtb* and *iscS-comp*. The strains *Mtb*, *Mtb*Δ*iscS*, and *iscS-comp* were treated with 1× MIC_90_ of (**C**) Mox (**D**) Rif, and (**E**) Inh, and survival over time was monitored by enumerating colony-forming units (CFUs). Data are presented as means ± SEM. (B to E) *P ≤ 0.05, **P ≤ 0.01, and ***P ≤ 0.001 calculated by unpaired two-tailed *t* test. ns, not significant.

Redox homeostasis, CCM, and respiration modulate *Mtb’s* response to anti-TB drugs (*44*). Furthermore, the ISC system in *E. coli* promotes killing by bactericidal antibiotics by providing iron for Fenton chemistry (*46*). In addition, ISC-mediated maturation of Fe-S clusters on respiratory complexes I and II promotes aminoglycoside killing by enabling respiration-dependent drug uptake (*47*). In the absence of ISC, cells switch to a less efficient SUF system for the maturation of Fe-S clusters on respiratory complexes, leading to defective uptake of aminoglycoside (*47*). However, a knowledge gap exists in our understanding of the role of Fe-S cluster biogenesis pathways in influencing lethality caused by anti-TB drugs. To begin understanding this, we measured the inhibition of *Mtb*Δ*iscS* growth by the clinically relevant anti-TB drugs rifampicin (Rif), isoniazid (Inh), moxifloxacin (Mox), and bedaquiline (Bdq). Using MABA, we determined MIC for Rif, Inh, Mox, and Bdq with WT *Mtb*, *Mtb*Δ*iscS*, and *iscS-comp* (Fig. 5A). *Mtb*Δ*iscS* exhibited two to fourfold lower MIC (therefore higher sensitivity) for Mox and Bdq (Fig. 5A). While *Mtb*Δ*iscS* showed no change in MIC for Rif, mutant growth was lowered by 84% at a subinhibitory concentration (8.8 nM) of Rif as compared to 37 to 67% inhibition of WT *Mtb* and *iscS-comp* (Fig. 5B).

We also measured the kill kinetics for Mox and Rif by colony-forming unit (CFU) analysis at various times after treatment with 1× MIC for WT *Mtb*. Compared to WT *Mtb*, *Mtb*Δ*iscS* displayed ~20- and 600-fold greater killing for Mox and Rif, respectively, at 10 days after exposure (Fig. 5, C and D). In contrast, *Mtb*Δ*iscS* exhibited higher MIC and resistance toward Inh (Fig. 5, A and E). One likely possibility is diminished levels of mycothiol in *Mtb*Δ*iscS*, which is known to cause Inh resistance in diverse mycobacterial species (*48*). Together, our data are in contrast with the current paradigm in bacteria, which indicates that Fe-S cluster biogenesis pathways promote killing by antibiotics (*46*). In *Mtb*, IscS reduces susceptibility toward anti-TB drugs.

### IscS protects *Mtb* from oxidative stress but not nitrosative stress

**Fig. 6.**
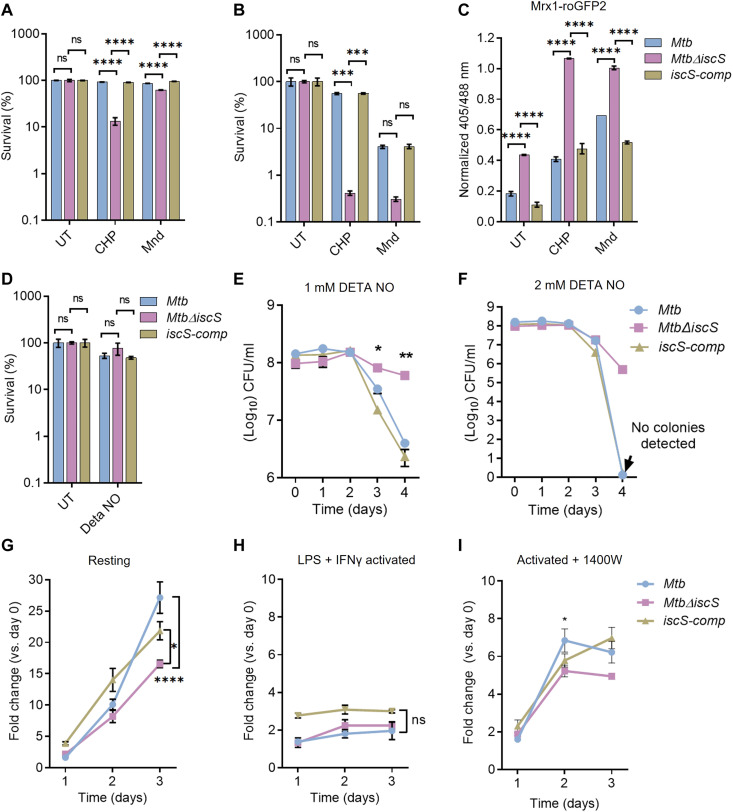
IscS provides resistance to oxidative stress but not nitrosative stress in *Mycobacterium tuberculosis *(*Mtb*). (**A** to **C**) Exponentially grown cells of *Mtb*, *Mtb*Δ*iscS*, and *iscS-comp* with or without Mrx1-roGFP2 were exposed to 80 μM cumene hydroperoxide (CHP) and 60 μM menadione (Mnd), followed by enumeration of colony-forming units (CFUs) for survival after 4 hours (A) and 24 hours (B). (C) Mrx1-roGFP2 ratio was measured at 4 hours after treatment with CHP or Mnd. (**D**) Survival of *Mtb*, *Mtb*Δ*iscS*, and *iscS-comp* after 24-hour exposure to nitric oxide (NO) donor diethylenetriamine (DETA) NO (1 mM). (**E** and **F**) Survival of *Mtb*, *Mtb*Δ*iscS*, and *iscS-comp* after repeated exposure to NO donor DETA NO (1 and 2 mM) was tracked for 4 days by CFU enumeration. Raw CFU values are plotted. (**G**) Naïve, (**H**) Interferon-γ (IFN-γ) + lipopolysaccharide (LPS)–activated RAW264.7, and (**I**) activated + 1400W (iNOS inhibitor)–treated macrophage were infected with *Mtb*, *Mtb*Δ*iscS*, and *iscS-comp* at an multiplicity of infection (MOI) of 1:2 for 4 hours, and intramacrophage survival was monitored over time by CFU enumeration. Data are presented as means ± SEM. (A to I) *P* ≥ 0.05, *P ≤ 0.05, **P ≤ 0.01, ***P ≤ 0.001, and ****P ≤ 0.0001 calculated by two-way analysis of variance (ANOVA) with Bonferroni’s multiple comparisons test. ns, not significant.

Fe-S cluster biogenesis systems also contribute to protection from oxidative and nitrosative stress in bacteria (*49*). In general, the Suf system maintains Fe-S cluster homeostasis during high demand (e.g., NO and iron starvation), whereas IscS coordinates housekeeping requirements for Fe-S clusters in diverse bacteria (*3*, *33*, *50*, *51*). The *Mtb* Suf system is required for Fe-S cluster biogenesis/repair both under low (standard growth conditions) and high demand (nitrosative stress) for Fe-S clusters (*10*, *11*). Although IscS seems important for optimal growth, its contribution in assisting *Mtb* to counteract stresses remains uncertain. Thus, we next examined the requirement for IscS in providing tolerance to stresses such as organic hydroperoxide (CHP; 80 μM), superoxide [menadione (Mnd); 60 μM], and NO donor [diethylenetriamine (DETA)/NO adduct (1 mM)]. WT *Mtb* and *Mtb*Δ*iscS* were treated with CHP and Mnd for 4 hours (Fig. 6A) and 24 hours (Fig. 6B), and survival was measured by CFU analysis. WT *Mtb* was unaffected at 4 hours, while 24-hour exposure led to a 20% decrease in survival (Fig. 6B). In contrast, 4-hour CHP and Mnd treatment was sufficient to reduce survival by 80 and 25% in *Mtb*Δ*iscS* relative to WT *Mtb*, respectively (Fig. 6A). At 24 hours after exposure, CHP and Mnd treatment resulted in ~99% lower survival as compared to WT *Mtb* (Fig. 6B). Using the Mrx1-roGFP2 biosensor, we confirmed that sensitivity to CHP or Mnd is associated with excessive oxidative stress in *Mtb*Δ*iscS*. We observed nearly complete oxidation of the biosensor in CHP- or Mnd-treated *Mtb*Δ*iscS* relative to WT *Mtb* within 4 hours of exposure (Fig. 6C). The *iscS-comp* strain showed a survival phenotype comparable to that of WT *Mtb* (Fig. 6, A to C). In contrast to oxidative stress, treatment of 1 mM DETA NO uniformly induces bacteriostasis in all three strains without any adverse effect on *Mtb*Δ*iscS* (Fig. 6D). Because NO’s ability to kill *Mtb* depends upon concentration and exposure time (*9*), we repeatedly challenged *Mtb* strains to 1 mM DETA NO every 24 hours for 4 days, and viability was assessed. We observed that WT *Mtb* and *iscS-comp* maintained survival until day 2 but displayed a gradual loss in viability at day 3 (10-fold) and day 4 (100-fold) after treatment (Fig. 6E). In contrast, *Mtb*Δ*iscS* retained viability throughout the experiment, indicating that the mutant resists nitrosative stress (Fig. 6E). A similar trend was observed upon repeated exposure to 2 mM DETA NO. In the case of WT *Mtb* and *iscS-comp*, 2 mM DETA NO resulted in a complete loss of viability at day 4 after treatment (Fig. 6F), corresponding to more than 5-log reduction in bacterial numbers (no colonies detected). In contrast, we observed only 100-fold killing of *Mtb*Δ*iscS* at day 4 after treatment (Fig. 6F).

To understand the relevance of in vitro findings to infection settings, we assessed survival of the mutant in macrophages. We infected resting and immune-activated RAW264.7 murine macrophages with *Mtb* strains at a multiplicity of infection (MOI) of 2 and monitored survival over time. The WT *Mtb* showed unrestricted growth in resting RAW264.7 over time. *Mtb*Δ*iscS* displayed a marginal (twofold) reduction in growth as compared to WT *Mtb* and *iscS-comp* at day 3 after infection (Fig. 6G). As expected, activation of macrophages prevents the multiplication of *Mtb* strains. *Mtb*Δ*iscS* showed survival comparable to WT *Mtb* and *iscS-comp* in activated macrophages (Fig. 6H). Because NO generated via inducible nitric oxide synthase (iNOS) is one of the main contributors of redox stress in *Mtb* inside immune-activated murine macrophages (*1*), these data agree with comparable survival of the mutant and WT *Mtb* in response to a bacteriostatic concentration of NO (single-dose 1 mM DETA NO) in vitro. We further verified the association between IscS and NO by treating RAW264.7 with the iNOS inhibitor 1400W (*52*) and examined the growth phenotype of *Mtb*Δ*iscS*. All three strains resumed proliferation in the presence of 1400W; however, the growth was marginally slow for *Mtb*Δ*iscS* compared to WT *Mtb* and *iscS-comp* (Fig. 6I).

### Disruption of IscS resulted in a hypervirulent mutant of *Mtb*

**Fig. 7.**
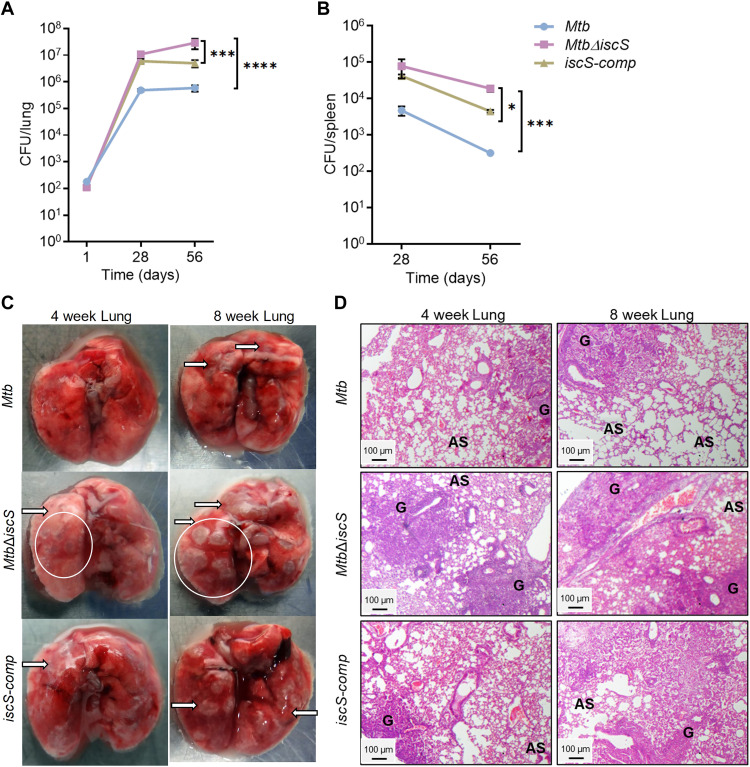
*Mtb*Δ*iscS* displays hypervirulence in mice. BALB/c female mice (*n* = 5) were given aerosol challenge with *Mycobacterium tuberculosis * (*Mtb*) , *Mtb*Δ*iscS*, *and iscS*-*comp* and assessed for survival in (**A**) lungs and (**B**) spleen at indicated time points. (**C**) The gross pathology of the infected lungs is shown after 4 and 8 weeks of infection. Black arrows show the granulomatous lesions formed upon *Mtb* infection. The white circles highlight the patches of lung consisting of multiple granulomatous lesions in the *Mtb*Δ*iscS*-infected lungs. (**D**) Hematoxylin and eosin–stained lung sections were imaged (at ×4 magnification) and analyzed for histopathology on *Mtb* infection. Changes in lung morphology are shown with formation of granuloma (G) and the normal alveolar spaces (AS). (A) *P* ≥ 0.05, *P ≤ 0.05, ***P ≤ 0.001, and ****P ≤ 0.0001 calculated by two-way analysis of variance (ANOVA) with Bonferroni’s multiple comparisons test. (B) *P ≤ 0.05 and ***P ≤ 0.001 calculated by unpaired two-tailed *t* test.

Depletion of Suf components (SufR and SufT) attenuated the survival and persistence of *Mtb* in vivo (*10*, *11*). However, the contribution of IscS to the pathogenesis of *Mtb* remains unknown. We examined the requirement for IscS in coordinating *Mtb’s* virulence in a mice model of experimental TB. BALB/c mice were infected with WT *Mtb*, *Mtb*Δ*iscS*, and *iscS-comp* via the aerosol route, and survival was determined by CFU counts at days 28 and 56 after infection. WT *Mtb* having a functional IscS showed increased bacterial burden until 28 days after infection, after which the lung bacillary load stabilized (Fig. 7A). In contrast with the poor survival of *Mtb*Δ*iscS* in vitro, the mutant showed higher survival and persistence in mice (Fig. 7, A and B). Lung bacterial burden in mice with *Mtb*Δ*iscS* was significantly higher (~50-fold) than that in those infected with the WT *Mtb* at 28 days after infection (Fig. 7A). The bacillary load in the lungs reached a maximum of ~100-fold (*P* ≤ 0.0001) higher for *Mtb*Δ*iscS* as compared to WT *Mtb*. A similar trend of elevated bacterial burden in the spleen of mice infected with *Mtb*Δ*iscS* as compared to WT *Mtb* was also detected (Fig. 7B).

Gross organ examination revealed that the lung surface of mice infected with *Mtb*Δ*iscS* showed discrete, well-circumscribed lesions (Fig. 7C) compared with poorly circumscribed and diffuse lesions in lungs of WT *Mtb-*infected mice. Histopathologic examination of the lungs of mice infected with WT *Mtb* revealed moderate granulomatous pneumonia with relatively smaller granulomas as compared to mild to severe granulomatous pneumonia characterized by multiple foci of granulomas with varying degrees of severity in the case of *Mtb*Δ*iscS* (Fig. 7D and fig. S11A). The *iscS-comp* strain showed partial attenuation of the hypervirulence phenotype in mice (Fig. 7A). Overall, the disruption of *iscS* in *Mtb* led to hypervirulence and abnormal granuloma formation in mice.

### Hypervirulence of *Mtb*Δ*iscS* is reduced by blocking NO-dependent *suf* induction

**Fig. 8.**
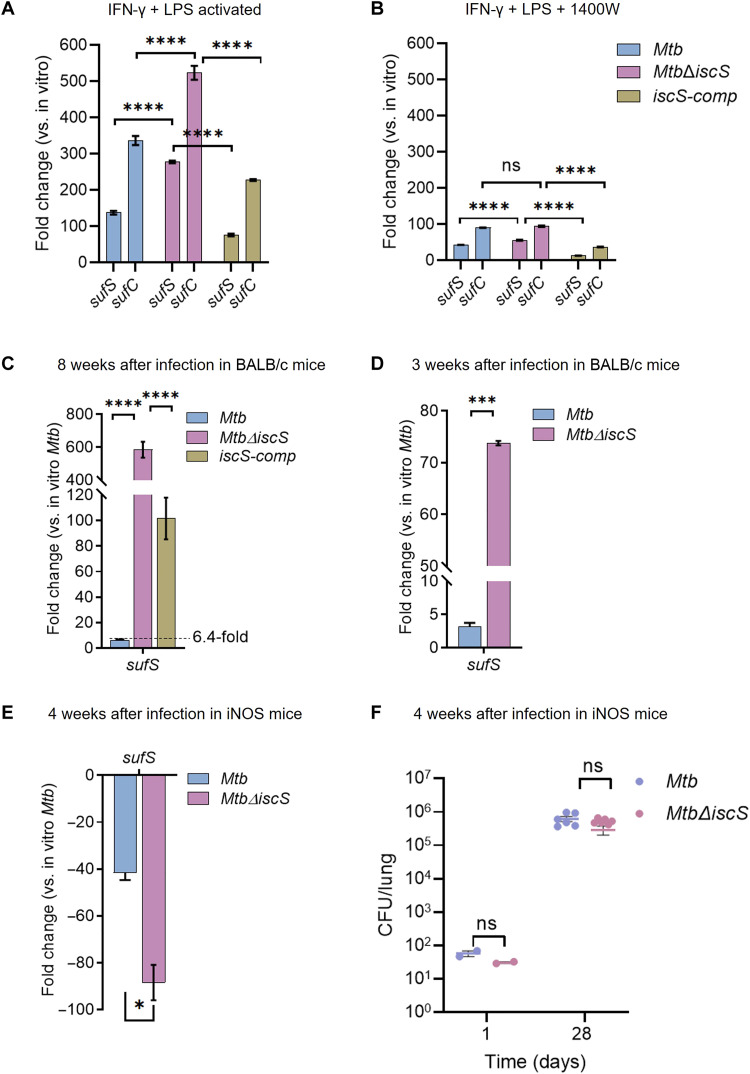
Nitric oxide (NO) induces the expression of Suf system and contributes to hypervirulence of *Mtb*Δ*iscS* in mice. Gene expression analysis by quantitative reverse transcription polymerase chain reaction (qRT-PCR) of bacterial Suf genes (*sufS* and *sufC*) in *Mtb*, *Mtb*Δ*iscS*, and *iscS-comp* isolated from infected RAW264.7 murine macrophages (**A**) Lipopolysaccharide (LPS) + interferon-γ (IFN-γ) activated and (**B**) activated + iNOS inhibitor (1400W) after 48 hours of infection. (**C**) Gene expression of *sufS* was analyzed in *Mtb*, *Mtb*Δ*iscS*, *and iscS*-*comp* isolated from mouse lung after 8 weeks of infection. (**D**) Similarly, transcript levels of *sufS* were determined in *Mtb* and *Mtb*Δ*iscS* isolated from 3-week–infected mouse lung. (A and B) Fold change in transcript levels is compared to that of respective strains grown under standard in vitro conditions and compared to in vitro *Mtb* (C to D). Data are presented as means ± SEM. (**E** and **F**) iNOS^−/−^ female mice (*n* = 6) were given aerosol challenge with *Mtb* and *Mtb*Δ*iscS* and assessed for survival in the lung after 4 weeks of infection. (F) Bacterial burden was determined by plating lung homogenates and colony-forming unit (CFU) enumeration. (E) The transcript levels of *sufS* were estimated in *Mtb* and *Mtb*Δ*iscS* isolated from 4-week–infected lungs of iNOS^−/−^ mice. (A, B, and F) ****P ≤ 0.0001, calculated by two-way analysis of variance (ANOVA) with Bonferroni’s multiple comparisons test. (C to E) Statistical significance was analyzed over untreated control by paired two-tailed *t* test (**P < 0.01 and ****P < 0.0001). ns, not significant.

ROI, RNI, iron starvation, and an intraphagosomal environment increase expression of the *suf* operon but not IscS in *Mtb* (*9*, *13*, *16*). We confirmed this observation by qRT-PCR and found that 5 mM H_2_O_2_ marginally induced (1.5- to 2.0-fold) *sufS* and *sufB* in WT *Mtb* and *Mtb*Δ*iscS* (fig. S12A). Notably, treatment with a bacteriostatic concentration of NO (0.5 mM DETA NO) induces an ~40- to 60-fold higher expression of *suf* genes (*sufS* and *sufB*) in WT *Mtb* and *Mtb*Δ*iscS* (fig. S12B). In contrast, NO reduces the expression of *iscS* (fig. S12B). However, at a bactericidal concentration of NO (2 mM DETA NO), the expression of *suf* genes was consistently higher in *Mtb*Δ*iscS* than in WT *Mtb* and *iscS-comp* (fig. S13A). We also performed qRT-PCR of *suf* genes on bacterial RNA isolated from immune-activated RAW264.7 infected with WT *Mtb* and *Mtb*Δ*iscS* in the presence or absence of iNOS inhibitor 1400W. Immune activation of macrophages induces the expression of *suf* genes by 100- to 300-fold in WT *Mtb* and 300- to 500-fold in *Mtb*Δ*iscS* (Fig. 8A). Inhibition of iNOS by 1400W uniformly represses the expression of *suf* genes to comparable levels in both strains (Fig. 8B).

On the basis of the above findings, we hypothesized that NO could be an environmental cue that induces *suf* genes, and the absence of IscS results in a compensatory, albeit unregulated increase in the *suf* operon, leading to a higher bacillary load with *Mtb*Δ*iscS* in mice. To test the above possibilities, we measured the expression of *sufS* as a proxy for the *suf* operon in bacterial RNA extracted from the lungs of mice infected with *Mtb* strains at 56 days after infection. We observed that the expression of *sufS* was 6.5-fold induced in WT *Mtb* derived from animal lungs compared to in vitro grown *Mtb* (Fig. 8C). Consistent with our hypothesis, the transcript of *sufS* was ~600-fold greater in *Mtb*Δ*iscS* than in vitro grown mutant bacilli (Fig. 8C). The *iscS-comp* strain showed a partial reduction in *sufS* transcript as compared to *Mtb*Δ*iscS* (Fig. 8C), which is consistent with partial complementation noted above. Induction of NO production was evident in mice after 14 to 24 days of infection with *Mtb* (*53*). Therefore, we quantified *sufS* expression in the lungs of infected mice at 21 days after infection. The expression of *sufS* was induced twofold in WT *Mtb*, whereas a 75-fold increase was detected in *Mtb*Δ*iscS* compared to in vitro grown conditions (Fig. 8D). To confirm that NO leads to the higher expression of the Suf system in mice, we infected a mouse strain lacking iNOS (iNOS^−/−^) with WT *Mtb* and *Mtb*Δ*iscS*, extracted bacterial RNA from lungs at 4 weeks after infection, and examined the expression of *sufS*. We found that the expression of *sufS* was 40- and 90-fold down-regulated in WT *Mtb* and *Mtb*Δ*iscS* derived from the lungs of iNOS^−/−^ mice as compared to in vitro grown bacteria, respectively (Fig. 8E). Consistent with these findings, we found that both the strains proliferated in mouse lungs without iNOS with no sign of hypervirulence exhibited by *Mtb*Δ*iscS* (Fig. 8F). The gross pathology of the lungs in both cases was comparable (fig. S14). While there was no difference in the bacillary burden for WT *Mtb* and *Mtb*Δ*iscS* in the lungs of iNOS^−/−^ mice, we observed that the colonies of *Mtb*Δ*iscS* recovered from the lung homogenates of iNOS^−/−^ mice were notably smaller than those of WT *Mtb*. The mutant colonies appeared only after 8 weeks of incubation as opposed to 2 to 3 weeks for WT *Mtb*. The reasons behind the delayed resumption of growth and small colony size of *Mtb*Δ*iscS* isolated from iNOS^−/−^ mice need future experimentation. Our data suggest that *Mtb* preferentially mobilizes the Suf system under Fe-S cluster–damaging conditions such as NO in vivo and the hypervirulence of *Mtb*Δ*iscS* is associated with NO-dependent overexpression of the *suf* operon during infection in mice.

### Depleting Suf system attenuates hypervirulence of *Mtb*Δ*iscS* during infection

We next sought to determine the impact of the *suf* operon on *Mtb*Δ*iscS*. Because the *suf* operon (*sufBCDSUT*) is essential (*7*), we interrupted its expression by anhydrotetracycline (ATc)–dependent CRISPRi-mediated depletion of *sufS* in WT *Mtb* and *Mtb*Δ*iscS* (fig. S15A) (*54*). Coexpression of *dCas9* (fig. S15B) and *sufS-*specific guide RNA (sgRNA) in response to ATc treatment uniformly reduced (~10-fold) the expression of *sufS* and downstream genes *sufU* and *sufT* in WT *Mtb* (*sufS-*KD) and *Mtb*Δ*iscS* (Δ*iscS-sufS* KD) (fig. S15, C and F). A ~10-fold reduction in *sufS* did not affect the survival of *sufS-*KD under aerobic growth conditions (fig. S15D) or in response to 5 mM H_2_O_2_ (fig. S15E). In contrast, Δ*iscS-sufS* KD grew slower than *Mtb*Δ*iscS* under aerobic conditions, and treatment with 5 mM H_2_O_2_ for 24 hours reduced the survival of Δ*iscS-sufS* KD by ~150- and 20,000-fold as compared to *Mtb*Δ*iscS* and WT *Mtb*, respectively (fig. S15, D and E). These data suggest that in the absence of IscS, *Mtb* uses the Suf system to sustain aerobic growth and survive H_2_O_2_ challenge. Expression of Suf system depends on a 4Fe-4S cluster containing the transcription factor SufR (*10*). Under Fe-S cluster–deficient conditions (e.g., NO and low iron), the cluster-less form of SufR (apo-SufR) derepresses the expression of the Suf system (*10*). We substantiated the compensatory role of the Suf system in the absence of IscS by examining the relative expression of the *suf* operon in WT *Mtb*, *Mtb*Δ*iscS*, *sufS*-KD, and Δ*iscS-sufS* KD by RNA-seq. Defects in both *iscS* and *sufS* (Δ*iscS-sufS* KD) induced the expression of the *suf* operon, which is higher than *Mtb*Δ*iscS* and marginally supersedes *sufS-*KD as compared to WT *Mtb* (fig. S15F). These findings demonstrate that Δ*iscS-sufS* KD is severely impaired in building Fe-S clusters, which results in defective aerobic growth and hypersensitivity to H_2_O_2_.

**Fig. 9.**
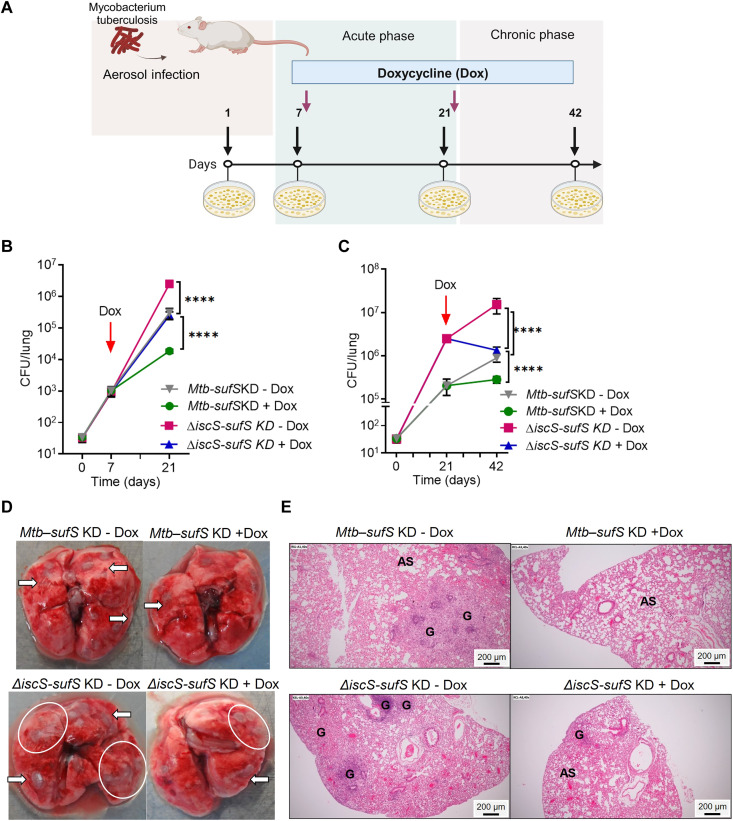
Overexpression of Suf system contributes to hypervirulence of *Mtb*Δ*iscS* in mice. (**A**) BALB/c mice (*n* = 5) were given aerosol challenge with *Mtb-sufS*KD and Δ*iscS-sufS*KD and divided into three groups of (i) no doxycycline treatment (−Dox), (ii) acute phase [doxycycline started at 7 days after infection (+Dox acute)], and (iii) chronic phase [doxycycline started at 21 days after infection (+Dox chronic)] (credit: BioRender.com). Post-infection animals were sacrificed from the (**B**) acute group at days 7 and 21 and (**C**) at days 21 and 42 from the chronic group, and colony-forming unit (CFU) per lung was measured. (**D**) Gross pathology of *Mtb-sufS*KD and Δ*iscS-sufS*KD–infected lungs at 42 days after infection. White arrows and white circles show the granulomatous lesions formed upon *Mycobacterium tuberculosis * (*Mtb*) infection. (**E**) Hematoxylin and eosin–stained lung sections were imaged (at ×40 magnification) and analyzed for histopathology on *Mtb* infection. Changes in lung morphology are shown with formation of granuloma (G) and the normal alveolar spaces (AS). (B and C) ****P ≤ 0.0001, calculated by two-way analysis of variance (ANOVA) with Bonferroni’s multiple comparisons test.

We infected BALB/c mice with the *sufS-*KD and Δ*iscS-sufS* KD strains by aerosol. As previously reported (*11*), we initiated SufSUT depletion in both strains at day 7 after infection to assess acute-phase phenotype and for the chronic phase at day 21 after infection by feeding doxycycline (Fig. 9A), a tetracycline derivative commonly used for gene silencing in vivo (*11*). The *sufS-*KD strain that retained IscS, but depleted SufSUT, showed the lowest lung bacillary load during both the acute and chronic phases of infection (Fig. 9, B and C). The Δ*iscS-sufS* KD mutant that lacked IscS and had depleted SufSUT exhibited a lung bacillary load intermediate between that of the severely attenuated *sufS-*KD and hypervirulent *Mtb*Δ*iscS* (Fig. 9, B and C). The bacterial burden of Δ*iscS-sufS* KD was similar with that of WT *Mtb* (Fig. 9, B and C). The gross and histopathological changes observed in the lungs of chronically infected mice were proportionate with the bacillary load observed (Fig. 9, D and E). The extent of pulmonary tissue destruction was highest in animals infected with *Mtb*Δ*iscS*, intermediate in WT *Mtb* and Δ*iscS-sufSKD* (score, ~15), and lowest in *sufS-*KD (score, 3.5) (fig. S16A). Thus, overinduction of the *suf* operon is likely responsible for the hypervirulence of *Mtb*Δ*iscS* in mice.

## DISCUSSION

*Mtb* expresses a nearly complete Suf system, thereby satisfying the demand for de novo Fe-S cluster assembly under normal growth conditions and repair in response to stress (*10*, *11*). Why *Mtb* retained a single gene (IscS) of the ISC system was initially unclear. The genetic, biochemical, and infection experiments described above show that IscS is required for maintaining redox balance, bioenergetics, antibiotic susceptibility, resistance to ROS, and survival inside macrophages. In the context of infection, deletion of IscS led to hypervirulence in mice, a phenotype linked to uncontrolled induction of the Suf system in response to NO. Our data indicate that *Mtb* uses the IscS and Suf systems to attain an intermediate degree of virulence that is critical for persistence.

Earlier transposon mutagenesis studies indicated that IscS is essential for *Mtb* (*55*). However, this is not the case, although IscS promotes optimal overall growth of *Mtb*. Previous studies were done at a subsaturating level, requiring statistical tools for downstream data processing to estimate essentiality. This affected the ability to assess the essentiality of many genes, including IscS, reliably. Consistent with this, a follow-up study used a completely saturated transposon library more authoritative cataloging of essential genes in *Mtb* and confirmed that *Mtb* IscS is not essential (*56*). Generally, Fe-S cluster biogenesis occurs during de novo assembly on the nascent apoproteins and repair of damaged Fe-S clusters (*57*). Similar to the IscS mutant of *E. coli* (*19*), assessment of *Mtb*Δ*iscS* indicated that most of the phenotypes exhibited by the mutant are likely due to inadequate de novo assembly rather than an inability to repair oxidized clusters. For example, when cells grow aerobically, the Fe-S clusters appear to be more sensitive to oxidative damage than in anaerobic conditions. Therefore, if IscS is functioning through the repair of oxidatively damaged Fe-S clusters, then the general growth defect of *Mtb*Δ*iscS* should show a greater dependence on oxygen. However, *Mtb*Δ*iscS* exhibited impaired survival under both aerobic and hypoxic conditions. Furthermore, *Mtb*Δ*iscS* accumulates ROS, which is known to convert [4Fe-4S]^2+^ clusters to [3Fe-4S]^1+^ clusters (*19*, *33*). The 3Fe-4S clusters can be repaired back to 4Fe-4S clusters upon scavenging of ROS without a requirement for either cysteine desulfurase or a sulfur source (*19*). In contrast, the reduction in ROS levels of *Mtb*Δ*iscS* by Thio did not restore the aerobic growth defect of the mutant. In the absence of IscS, *Mtb* likely accumulates incomplete Fe-S clusters that lack sulfur atoms, which differ from oxidatively damaged Fe-S clusters. Consistent with this idea, the pool of unincorporated intracellular iron was elevated in *Mtb*Δ*iscS*. Free iron could cause ROS accumulation in *Mtb*Δ*iscS* via the Fenton reaction (*26*). However, in contrast to our expectations, ROS accumulation and the slow growth defect were accentuated upon treatment of *Mtb*Δ*iscS* with an iron chelator. These results suggest that ROS increase in *Mtb*Δ*iscS* is not a consequence of free iron. We have recently shown that increased NADH/NAD^+^ ratio and NADH-reductive stress are critical for iron-mediated ROS surge (*58*). *Mtb*Δ*iscS* showed neither an increased NADH pool nor higher NADH/NAD^+^ poise, which suggests that iron accumulation is unlikely to drive ROS generation in the mutant. Because iron is a cofactor for several antioxidant enzymes (e.g., superoxide dismutase and catalase) (*59*, *60*), ROS accumulation could be due to disruption of their activities upon iron limitation. A possible explanation for the elevated oxidative stress upon iron chelation in *Mtb*Δ*iscS* is an impairment of the metabolic and respiratory functions, mainly due to reduced synthesis of Fe-S clusters for enzymes of the TCA cycle and respiratory chain. Similarly, dysfunction of mechanisms that prevented the assembly of mitochondrial Fe-S cluster led to ROS accumulation and DNA damage (*61*).

The slow-growth phenotype of *Mtb*Δ*iscS* was expected because several metabolic enzymes and respiratory complexes contain Fe-S clusters. Consistent with this, *Mtb*Δ*iscS* had diminished glycolytic, PPP, and TCA cycle intermediate pool. These results aligned well with impaired oxygen consumption, reduced glycolysis, and reductant levels (NADH/NADPH) in *Mtb*Δ*iscS*. Surprisingly, despite a previous study showing ~50% reduction in aconitase and succinate dehydrogenase activities in *Mtb*Δ*iscS* (*17*), the respective substrates and products of these enzymes remain unaltered in the mutant. One possibility is that the residual activity of aconitase and succinate dehydrogenase is due to the Suf system. Alternatively, in the absence of IscS, *Mtb* could attempt to restore metabolism by operating the TCA cycle in the reverse direction. This appears to be partially successful and could have resulted in the restoration of citrate, α-KG, and oxaloacetate in *Mtb*Δ*iscS*. However, accumulation of fumarate and reduction in succinate and malate indicate the impaired activity of a reverse TCA cycle enzyme fumarate reductase, which contains an Fe-S cluster (*39*), and a canonical TCA enzyme fumarate hydratase in *Mtb*Δ*iscS*, respectively. Our RNA-seq data confirm that genes expressing fumarate reductase are down-regulated in *Mtb*, which could be another reason why fumarate reductase activity is likely more affected than succinate dehydrogenase.

The gene encoding fumarate hydratase was unaffected in the mutant. The fumarate hydratase catalyzes the stereospecific hydration of fumarate to malate and is essential for *Mtb’s* survival in vitro and in vivo (*62*). At least two possible mechanisms could link fumarate hydratase with redox balance and Fe-S cluster biogenesis in *Mtb*. First, fumarate hydratase maintains redox homeostasis by preventing fumarate accumulation and subsequent covalent modification of mycothiol and thiol-containing proteins via succination in *Mtb* (*62*). Second, in mammalian cells, proteins involved in Fe-S cluster biogenesis and respiration are sensitive targets of succination, directly linking fumarate hydratase functionality with Fe-S cluster biogenesis and bioenergetics (*63*). Additional experiments are needed to determine whether IscS activity is regulated by fumarate hydratase and succination in *Mtb*.

While any of the above Fe-S cluster–dependent mechanisms could have contributed to the slow growth rate of *Mtb*Δ*iscS*, other cellular functions requiring IscS as a sulfur donor, such as the thiolation of tRNA, might contribute to these metabolic phenotypes. In this context, IscS exists in an operon with *trmU*, which encodes 2-thiouridylase. This enzyme is known to catalyze the formation of 2-thiourdine *in E. coli* tRNA (*64*). While IscS physically associates with TrmU, deletion of *trmU* neither reduced aconitase activity nor affected the survival of *Mtb* under H_2_O_2_ stress (*17*). However, our transcriptomics data suggest that *trmU* expression remained down-regulated in *Mtb*Δ*iscS* and showed only partial restoration in *iscS-comp*, which could explain incomplete complementation of the mutant phenotypes in some of our assays. A more careful examination is needed to decipher the role of IscS and TrmU in the sulfur metabolism of *Mtb*.

Our metabolomics and transcriptomics data provide insight into why *Mtb* harbors a truncated ISC system in the backdrop of a complete SUF pathway. Both IscS and Suf systems are expressed and contribute to enzymatic activities of the Fe-S cluster proteins aconitase and succinate dehydrogenase under normal growth conditions (*11*, *17*). However, the absence of IscS mainly affected metabolites unrelated to Fe-S cluster enzymes (e.g., glycolysis and PPP), except for the TCA cycle enzyme fumarate reductase. In contrast, depletion of SufT resulted in an altered pool of many metabolites directly or indirectly dependent on Fe-S cluster enzymes (*11*). These observations explain why the Suf system is essential for the growth of *Mtb,* whereas disruption of IscS only slows growth under aerobic conditions. Because the expression of the Suf system was maintained in *Mtb*Δ*iscS*, the residual metabolic, redox, and bioenergetic activities in the mutant could be due to the action of the Suf system.

The connection between the Suf system and IscS becomes clearer under conditions that highly induce the *suf* operon. NO treatment induces an elevated and long-lasting expression of the *suf* operon in *Mtb* (*9*), which remained comparable in *Mtb*Δ*iscS* (Fig. 8B). In contrast, hypoxia represses the *suf* operon (*65*). In line with this, nitrosative stress did not affect *Mtb*Δ*iscS*, whereas hypoxic conditions reduced survival of *Mtb*Δ*iscS*. In general, mild oxidizing conditions, such as low concentrations of H_2_O_2_ or O_2_, oxidize a [4Fe-4S]^2+^ cluster to a [3Fe-4S]^+^ cluster that can be repaired back to the original [4Fe-4S]^2+^ under reducing conditions in the presence of ferrous ion (*6*). For these environmental situations, basal expression of both IscS and Suf is sufficient. However, for proteins whose clusters are degraded beyond the [3Fe-4S]^+^ state or to apo-form or dinitrosyl-iron dithiol complex (DNIC), wherein the sulfide ligands of the 4Fe-4S clusters were displaced by NO to form [Fe-(NO)_2_], sulfur atoms must be supplied by inducing de novo–style Fe-S cluster biogenesis pathways for rebuilding the Fe-S clusters (*6*, *10*). This seems to be the case with *Mtb*, wherein the expression of the *suf* operon is repressed by a 4Fe-4S cluster containing SufR (holo-SufR) under standard aerobic growth conditions. At the same time, cluster-less SufR (apo-SufR) derepresses Suf expression (*10*). The 4Fe-4S cluster of SufR is resistant to mild oxidizing conditions (O_2_) (*10*). NO or high molar concentrations of H_2_O_2_ rapidly damage 4Fe-4S of SufR to DNIC or apo-form, respectively, resulting in the de-repression of the *suf* operon in vitro and in vivo (*10*). In this context, data using iNOS inhibitor and iNOS^−/−^ mice confirm the induction of the *suf* operon in response to NO during infection of macrophages and mice. The absence of a survival defect of *Mtb*Δ*iscS* when inside the immune-activated murine macrophages suggests that the NO/H_2_O_2_-driven induction of *suf* operon might compensate for the loss of IscS. Consistent with this observation, we showed that residual survival of *Mtb*Δ*iscS* under aerobic or H_2_O_2_ stress is due to the expression of the *suf* operon.

Our phenotypic data suggest that IscS and Suf systems work in distinct ways. *Mtb* requires IscS system more under hypoxic conditions where the demand for Fe-S clusters is minimal. As the demand for Fe-S cluster increases, such as during growth in an oxygen-rich environment, H_2_O_2_, and upon exposure to NO stress, *Mtb* gradually shifts its reliance from IscS to Suf for maintaining Fe-S cluster homeostasis and survival. The *E. coli* Isc system is generally poisoned with as low as 1 μM H_2_O_2_, whereas the Suf system remains resistant to oxidative stress (*6*). Similar differences in sensitivity to oxidants are expected for *Mtb* IscS and Suf systems (*10*, *11*, *17*). However, the artificial overproduction of IscS suppresses the growth defect of *Mtb*Δ*iscS* and renders the mutant more resistant to H_2_O_2_ than WT *Mtb* (*17*). Thus, in principle, the induction of SUF/ISC pathways elevates the total cellular capacity for cluster assembly during high-demand conditions rather than being intrinsically more resistant to H_2_O_2_ or NO. Agreeing with this, overexpression of the Suf system in *Mtb*Δ*iscS* inside murine lungs increased bacillary load and pathological defects more than WT *Mtb*.

During infection in mice, *Mtb* is exposed to a more potent RNI-peroxynitrite that primarily damages Fe-S clusters with a high-rate constant (~1.0 × 10^10^ M^−1^ s^−1^) (*66*). While NO largely induces the Suf operon to the same level in WT *Mtb* and *Mtb*Δ*iscS* in vitro, peroxynitrite exposure during murine infection could have caused additional damage to Fe-S cluster proteins including SufR in the mutant, leading to more induction of Suf system in *Mtb*Δ*iscS* and hence hypervirulence. This agrees with our findings showing both the loss of *suf* inducibility and hypervirulence phenotype of *Mtb*Δ*iscS* in iNOS^−/−^ mice lacking the ability to produce peroxynitrite. Consistent with a dominant role of the Suf system during stress, organisms that are routinely exposed to H_2_O_2_ (e.g., lactic acid bacteria, *Enterococcus faecalis*, *Xylella fastidiosa,* and chloroplasts) rely upon Suf rather than ISC for cluster assembly (*6*, *67*, *68*). While true for other organisms, *Mtb’s* persistence depends upon an adequate response toward oxidative/nitrosative stress and hypoxia. Therefore, the importance of IscS in mediating the response to hypoxia in vitro and suppressing hypervirulence by adjusting Suf expression in vivo provides the biological significance of IscS in the persistence of *Mtb*. This notion must be tested in animal models (e.g., guinea pigs and nonhuman primates) (*69*), exposing *Mtb* to hypoxic lesions during infection.

## MATERIALS AND METHODS

### Bacterial strains and culture conditions

The *Mtb*Δ*iscS* and *iscS-comp* (*pHsp_60_*) strains were gifts from S. T. Cole (Pasteur Institute, Paris, France). The *iscS-comp* was constructed for this study by cloning 500–base pair (bp) upstream region of *iscS* into the integrative plasmid pCV125. *Mtb-*roGFP2 strain was generated by transforming *Mtb* strains with an *E. coli*– mycobacterial shuttle vector, pMV762-Mrx1-roGFP2 (biosensor construct), with hygromycin resistance gene as selection marker (*22*). All mycobacterial strains were grown in Middlebrook 7H9 broth (Becton, Dickinson and Company, USA) liquid medium supplemented with 0.2% glycerol, 0.05% Tween 80, and ADS (0.5% albumin, 20% dextrose, and 0.085% NaCl) or OADC (ADS plus 0.05% oleic acid and 0.004% catalase) with shaking at 180 rpm in a shaker incubator (Lab Therm LT-X, Kuhner, Basel, Switzerland) or on 7H11 agar (solid medium) supplemented with ADS or OADC at 37°C. As per the requirement, antibiotics were added to the culture medium at a concentration of kanamycin (KAN; 25 μg/ml) (Amresco, USA) and hygromycin (HYG; 50 μg/ml) (MP Biomedicals, Santa Ana, CA). *E. coli* DH5α strains were grown in LB broth/agar (Himedia, India) with antibiotic concentrations of KAN (50 μg/ml) and HYG (100 μg/ml).

### Measurement of *E*_MSH_ using the Mrx1-roGFP2 redox biosensor

The intra-mycobacterial *E*_MSH_ (defined as the standard reduction potential of the MSH_reduced_/MSSM_oxidized_ redox couple) determination during in vitro growth of *Mtb*, *Mtb*Δ*iscS*, and *iscS-comp* was performed as in (*22*). Briefly, bacterial cultures expressing Mrx1-roGFP2 were treated with 5 mM *N*-ethylmaleimide (Sigma-Aldrich, St. Louis, MO) for 5 min at room temperature followed by 4% paraformaldehyde (PFA) fixation (Himedia, Mumbai, India) for 1 hour at room temperature. Bacteria were analyzed using a FACSVerse flow cytometer (BD Biosciences, San Jose, CA). The *E*_MSH_ was calculated using the Nernst equation as described previously in (*22*). The biosensor response was quantified by measuring the fluorescence ratio at a fixed emission (510 nm) on excitation at 405 and 488 nm. The data obtained were analyzed with the BD FACSuite software. These ratiometric data were normalized to measurements of cells treated with 10 mM CHP (Sigma-Aldrich, St. Louis, MO), giving maximal oxidation of the biosensor, and 20 mM dithiothreitol (Sigma-Aldrich, St. Louis, MO), yielding a readout of maximal reduction of the biosensor. Ten thousand events per sample were analyzed. Biosensor response was measured in a similar way for bacterial cells exposed to ROS (80 μM CHP or 60 μM Mnd) after 4-hour treatment.

### ROS measurement using CellROX deep red

Exponentially growing cultures of *Mtb*, *Mtb*Δ*iscS*, and *iscS-comp* were taken for measurement of intracellular ROS. According to the manufacturer’s instructions, CellROX Deep Red (Invitrogen, Waltham, MA) was added to a final concentration of 5 μM, and cells were stained by incubating on a rocker for 30 min at 37°C. The cells were then washed with 1× phosphate-buffered saline (PBS; pH 7.4) to remove residual dye by centrifugation (5000 rpm for 5 min). Last, cells were resuspended in 300 μl of PBS and fixed using 4% PFA for 1 hour at room temperature. Fluorescence was measured using a BD FACSVerse flow cytometer (BD Biosciences, San Jose, CA) at a fixed emission maxima of 670 nm allophycocyanin (APC) channel) after excitation with a red laser (640 nm) for 10,000 events per sample. No autofluorescence was observed.

For determination of intracellular ROS in the presence of Bip and Thio, exponentially grown cultures at an initial OD_600_ (optical density at 600 nm) of 0.6 (10 ml) were treated with 250 μM Bip and 10 mM Thio separately and incubated for 24 hours in a shaker incubator (180 rpm, 37°C). This was followed by the above procedure for CellROX Deep Red staining and ROS measurement.

### Cellular iron estimation

Intracellular iron levels were measured using the ferrozine-based colorimetric assay described previously (*70*, *71*). Briefly, exponentially grown cultures of *Mtb* strains (OD_600_ ~ 0.8) were harvested by centrifugation and washed twice with ice-cold PBS. The cell pellets were resuspended in 1 ml of 50 mM NaOH and lysed using a bead beater (MP Biomedicals, Santa Ana, CA). HCl (10 mM, 300 μl) was added to the cell lysate samples (300 μl), followed by the addition of the Fe-detection reagent (6.5 mM ferrozine, 6.5 mM neocuproine, 1 M ascorbic acid, and 2.5 M ammonium acetate in water) (90 μl). This reaction mix was incubated for 30 min at 37°C, followed by reading the absorbance of the samples at 562 nm using a microplate reader (Versa-Max; Molecular Devices, San Jose, CA). The cellular free iron concentration was equated by plotting the absorbance values against a standard curve of FeCl_3_ concentration gradient and readouts normalized to the protein content of the respective samples. Protein concentration was estimated using a Pierce bicinchoninic acid (BCA) protein assay kit (Thermo Fisher Scientific, Rockford, IL).

### Determination of bacterial survival

The number of viable bacilli was determined after treatment with 250 μM iron chelator Bip or 10 mM ROS scavenger Thio by removing aliquots from the cultures followed by preparation of 10-fold dilutions. Twenty microliters of dilutions was trailed on 7H11 agar plates. Plates were incubated for 3 to 5 (for *Mtb*Δ*iscS*) weeks at 37°C, followed by CFU enumeration.

### Establishment of hypoxia

To determine the viability of *Mtb*, *Mtb*Δ*iscS*, and *iscS-comp* under hypoxic conditions, bacterial cultures (OD_600_ = 0.1) were injected into vacutainer tubes (Becton Dickinson, Franklin Lakes, NJ) followed by incubation at 37°C for 12 to 16 days (*58*, *72*). A marker for the achievement of hypoxia was the decoloration of MB (final concentration of 1.5 μg/ml) (Sigma-Aldrich, St. Louis, MO) in the culture medium. Once hypoxia was established, cells were harvested for determination of bacterial survival by CFU.

### Growth curves for *Mtb* strains

Freezer stocks of *Mtb* strains were revived in 7H9-OADC broth with 1:10 dilution and grown for 1 week. These cultures were subcultured and inoculated in 7H9-ADS at OD_600_ ~ 0.05 and incubated at 37°C at 180 rpm, and growth was tracked for 8 to 9 days by measuring the OD_600_ until the cultures reached a stationary phase.

### Aconitase activity

The aconitase activity was measured by monitoring the disappearance of *cis*-aconitate at wavelength (λ) 240 nm in an ultraviolet spectrophotometer (Thermo Fisher Scientific, Biomat 3S, USA) as described (*11*). One unit of aconitase activity is defined as 1 μmol of *cis*-aconitate formed or converted per minute. Reaction mixtures for aconitase assay contained 25 mM tris-HCl (pH 8.0), 100 mM NaCl, and 50 μg of *Mtb* cell lysates in 1 ml of reaction volume. Reaction was initiated by adding 0.15 mM *cis*-aconitate and monitored by following the disappearance of *cis*-aconitate at λ 240 nm after every 15 s for 30 min. Absorbance at λ 240 nm was plotted against time. Aconitase activity was calculated from linear portion of the curve in initial 5 min. An extinction coefficient of 3500 M^−1^ cm^−1^ was used to calculate the rates.

### Metabolite extraction and analysis

Metabolite extraction from all *Mtb* strains was performed as outlined in (*11*). Briefly, exponentially growing *Mtb*, *Mtb*Δ*iscS*, and *iscS-comp* cultures (OD_600_ ~ 0.6) were quenched with four volumes of 60% methanol for 5 min (maintained at −45°C) in a dry ice-methanol bath, followed by centrifugation at 4000 rpm for 5 min (at −5°C). The pellet was resuspended in 700 μl of 60% methanol (maintained at −45°C) and centrifuged at 4000 rpm for 5 min. The pellet obtained was resuspended in 1 ml of 75% ethanol and incubated at 80°C for exactly 3 min, with intermittent mixing at 1.5-min intervals, followed by incubation on ice for 5 min and centrifugation at 13,000 rpm for 15 min. The final supernatant was lyophilized and then stored at −80°C until further analysis.

Steady-state levels of metabolites were analyzed using methods described in (*73*). Briefly, extracted metabolites were first separated using a Synergi 4-μm Fusion-RP 80 Å (150 × 4.6 mm, Phenomenex) LC column on the Shimadzu Nexera ultrahigh-performance LC system. For TCA intermediates, derivatization was done before separation. The following solvent systems were used: 0.1% formic acid in water (solvent A) and 0.1% formic acid in methanol (solvent B) for amino acids, nucleotides, and TCA metabolites and 5 mM ammonium acetate in water (solvent A) and 100% acetonitrile (solvent B) for sugar phosphates. The flow parameters used were as described in (*73*). The mass spectrometer used was AB Sciex Qtrap 5500, and data acquisition was made by Analyst 1.6.2 software (Sciex). Amino acids and TCA intermediates were detected in positive polarity, while sugar phosphates were detected in negative polarity mode. The parent and daughter ion masses for each metabolite are given in table S3. The data were analyzed by calculating the area under the obtained peaks by using Multi Quant (version 3.0.1) software.

### Estimation of NAD^+^, NADH, NADP^+^, and NADPH

*Mtb* strains were grown to OD_600_ ~ 0.8 and harvested for the detection of pyridine nucleotide levels by a redox-cycling assay as described in (*74*, *75*). NADPH and NADP^+^ concentrations were normalized to the protein contents of NADH and NAD^+^ extracts using BCA protein assay.

### OCR and ECAR measurements

To estimate the basal OCR and ECAR, *Mtb*, *Mtb*Δ*iscS*, and *iscS-comp* cultures of OD_600_ ~ 0.6 were briefly (24 hours) incubated in 7H9 medium containing the nonmetabolizable detergent tyloxapol (MP Biomedicals, Santa Ana, CA) and devoid of ADS or any carbon source. These cultures were then harvested, and single-cell suspensions of bacteria were prepared by passing five times through a 26-gauge syringe needle followed by centrifugation at 100*g* for 1 min. Cells (2 × 10^6^ per well) were seeded into the wells of an XF cell culture microplate (Agilent/Seahorse Biosciences, Santa Clara, CA) coated with Cell-Tak (Corning, Corning, NY). Measurements were carried out using a Seahorse XFp analyzer (Agilent Technologies, CA) with unbuffered 7H9 as the assay media (pH 7.35; lacking disodium phosphate and monopotassium phosphate). Basal readings were taken for an initial 18 min, followed by an injection of d-glucose (10 mM), giving glucose-induced respiration rates. The maximum rate of respiration was achieved by the addition of two consecutive doses of 2 and 8 μM CCCP (Sigma-Aldrich, St. Louis, MO). Readouts were normalized to the number of bacteria seeded.

### RNA isolation, amplification, and library preparation for RNA-seq

*Mtb* strains grown to an OD_600_ of 0.4 were harvested in 5 M guanidinium thiocyanate (GTC) buffer (containing 1% 2-mercaptoethanol, 0.5% sarcosyl, and 0.5% Tween 80) for total RNA extraction. Total RNA was extracted using a FastRNA Pro Blue kit (MP Biomedicals, USA) and further purified with RNeasy spin columns (Qiagen, USA) as described (*14*). The concentration and quality of extracted RNA were checked spectrophotometrically using NanoDrop ND-1000 (Thermo Fisher Scientific). Total RNA was then enriched for mRNA by depletion of 16*S* and 23*S* rRNA using the MICROBExpress Kit (Life Technologies, USA), and the concentration of the ribo-depleted RNA was quantified by the QuBit RNA HS Assay Kit (Life Technologies). Fifteen nanograms of mRNA per sample was taken for fragmentation and random priming, followed by first and second cDNA synthesis and library preparation using the NEBNextUltra II Directional RNA Library Prep Kit for Illumina (New England Biolabs), according to the manufacturer’s protocol. The library size distribution and quality were assessed using a high-sensitivity DNA chip (Agilent Technologies). Last, equimolar quantities of all libraries were pooled and sequenced in a high-throughput run on the Illumina HiSeq 2500 sequencer (*14*).

### Differential gene expression and statistical analysis for RNA-seq

The reference genome sequence (.fna) and annotation (.gff) files for the *Mtb* H37Rv strain (accession number: NC_000962.3) were downloaded from the National Center for Biotechnology Information (NCBI) ftp website (“ftp.ncbi.nlm.nih.gov“). The annotation file format (.gff) was changed to .bed using an in-house Python script. Sequencing-based raw reads obtained (as .fastq) were checked for quality using FastQC software (version v0.11.5; http://www.bioinformatics.babraham.ac.uk/projects/fastqc). BWA (version 0.7.12-r1039) (*76*) was used to index the reference genome. Reads having raw read quality of ≥20 were aligned using the BWA aln -q option. SAMTOOLS (version 0.1.19-96b5f2294a) (*77*) was used to filter out the multiple mapped reads. Read count per gene was calculated using BEDTOOLS (version 2.25.0) (*78*) with the annotation (.bed) file. The normalization and DEG analysis for the data were carried out using edgeR, as mentioned previously (*79*). DEGs were determined on the basis of the cutoff: absolute fold change ≥ 1.5 and FDR ≤ 0.05.

For overlap analysis of DEGs with the processed data of other studies, the significance of gene number overlap was determined by Fisher’s exact test on a two-by-two contingency table. The universal set of the genes was calculated on the basis of the intersection of total genes analyzed in the studies with our study. If this information was not available, then we took 3975 genes as the universal set (table S2).

### Bacterial stress survival assay

Exponentially growing *Mtb*, *Mtb*Δ*iscS*, and *iscS-comp* (OD_600_ ~ 0.6) cultures were diluted to an OD_600_ of ~0.15 and exposed to 80 μM CHP, 60 μM Mnd (Sigma-Aldrich, St. Louis, MO), 1 mM DETA-NO (Cayman Chemical, USA) and incubated at 37°C in a shaker incubator. After 4 and 24 hours of treatment, cells were serially diluted and plated on 7H11-ADS plates. Colonies were enumerated after an incubation of 3 to 5 weeks at 37°C.

### Macrophage preparation and *Mtb* infection

RAW264.7 murine macrophage cell line was used for the ex vivo infection study. This cell line was acquired from the American Type Culture Collection (Manassas, VA) and tested negative for mycoplasma contamination using the DE-MycoX Mycoplasma PCR Detection Kit (CELLclone, catalog no. GX-E-250). *Mtb* infection was performed in either naïve or activated macrophages. Macrophage activation was achieved by treating RAW264.7 cells with interferon-γ (IFN-γ) (30 ng/ml; PeproTech, catalog no. 315-05) and *E. coli* lipopolysaccharide (LPS) (10 ng/ml; Sigma-Aldrich, catalog no. L2630) for 16 hours. These activated macrophages were separately treated with a specific inhibitor of iNOS, 1400W dihydrochloride (25 μM) (Sigma-Aldrich, catalog no. W4262). Macrophage cells were infected with *Mtb*, *Mtb*Δ*iscS*, and *iscS-comp* at an MOI of 1:2 for 4 hours, followed by washing thoroughly with warm PBS (137 mM NaCl + 2.7 mM KCl + 10 mM Na_2_HPO_4_ + 1.4 mM KH_2_PO_4_) and Dulbecco’s modified Eagle’s medium (DMEM; CELLclone) to remove all extracellular bacteria and finally added fresh media [DMEM + 10% fetal bovine serum (MP Biomedicals, catalog no. 092916754)] with or without IFN-γ and incubated at 37°C with 5% CO_2_. To determine bacterial survival after infection at different time points, infected cells were lysed in 0.06% SDS in PBS. Lysates were serially diluted and plated on 7H11-OADC agar plates. Plates were incubated at 37°C until colonies appeared for CFU enumeration.

### Determination of MIC and antibiotic susceptibility assay

MIC was evaluated by a MABA in a 96-well plate format (*58*). Exponentially growing *Mtb* strains (OD_600_ ~ 0.6) were harvested to prepare 1 × 10^6^ cells/ml density culture. Approximately 1 × 10^5^ bacteria were added per well in a total volume of 200 μl of 7H9 + ADS medium containing a gradient of drug concentrations. Controls for the assay consisted of wells lacking *Mtb* for a media background and wells devoid of a drug for maximum bacterial growth. Following an incubation period of 5 days at 37°C, 30 μl of 0.02% resazurin (Sigma-Aldrich, catalog no. R7017) was added, and plates were incubated for another 24 hours at 37°C. Fluorescence intensity was detected using a SpectraMax M3 plate reader (Molecular Devices, San Jose, CA) in a bottom-reading mode with excitation at 530 nm and emission at 590 nm. Percent inhibition for respective antibiotics was derived from the relative fluorescence values compared to the untreated control. MIC was considered the minimum antibiotic concentration that yielded at least 90% reduction in fluorescence compared to the untreated growth control. Antibiotic concentration ranges were as follows: Bdq (1.8 to 0.005 μM), Mox (1 to 0.003 μM), Rif (1.13 to 0.003 μM), and Inh (7.3 to 0.021 μM).

### Antibiotic kill kinetic assay

Exponentially growing cultures of *Mtb* strains were brought to a density of 1 × 10^6^ CFU/ml and incubated with or without 1× MIC_90_ (for *Mtb*) of antibiotics: Mox, Rif, and Inh in 10-ml cultures for a period of 10 days in a shaker incubator at 37°C. The survival kinetics of the bacteria was monitored by plating for viable cells on days 0, 1, 3, 5, 7, and 10 after treatments. Following incubation at 37°C for 3 to 5 weeks, viable colonies were enumerated.

### In vivo infection experiments

For the mice infection model, 5- to 6-week-old female BALB/c mice (*n* = 5 per group) were infected via an aerosol route with approximately 100 bacilli per mouse with *Mtb*, *Mtb*Δ*iscS*, and *iscS-comp* strains using a Madison chamber by aerosol generation. At indicated times after infection, mice were sacrificed, and the lungs and spleens were harvested for determination of bacillary load and tissue histopathology analysis as described (*14*). The bacillary load was quantified by plating serial dilutions of tissue homogenates on 7H11-OADC agar plates supplemented with lyophilized BBL MGIT PANTA antibiotic cocktail (polymyxin B, amphotericin B, nalidixic acid, trimethoprim, and azlocillin; as supplied by BD Biosciences, USA). Colonies were enumerated after 4 weeks of incubation at 37°C. Pathological analyses were represented as granuloma scores as described previously (*14*).

Mice infection experiment with the SufS-KD strains was performed similarly, where mice were divided into six groups, *Mtb-sufS* KD − Dox (without doxycycline), *Mtb-sufS* KD + Dox acute phase (doxycycline started after 7 days of infection), *Mtb-sufS* KD + Dox chronic phase (doxycycline started after 21 days of infection), Δ*iscS-sufS* KD − Dox (without doxycycline), Δ*iscS-sufS* KD + Dox acute phase (doxycycline started after 7 days of infection), and Δ*iscS-sufS* KD − Dox chronic phase (doxycycline started at 21 days after infection). Doxycycline (1 mg/ml) was given in drinking water with a 5% sucrose solution. Water feeders were light-protected and replaced twice a week (*11*).

To determine the role of NO in *Mtb* infection and pathogenesis, 5- to 7-week-old female iNOS^−/−^ mice (*n* = 6) were infected with approximately 100 bacilli per mouse with *Mtb* and *Mtb*Δ*iscS* strains using a Madison chamber by aerosol generation. Mice were humanely sacrificed, and the lungs were harvested for determination of bacillary load. The bacillary load was quantified by plating serial dilutions of tissue homogenates as described above. All mice for the above experiments were obtained from the institute Central Animal Facility (CAF).

### Generation of conditional knockdowns using CRISPRi strategy

Construction of SufS-KD strains was carried out using the CRISPRi technology as described in (*54*). Briefly, an inactive form of *Streptococcus pyogenes Cas9* enzyme harboring mutations D10A and H820A (*dCas9*) resulting in nuclease inactivity offers the ability to control targeted gene expression transiently or stably without altering the genomic sequence. dCas9 was expressed in the integrative plasmid system pRH2502 from a TetR-regulated uvtetO promoter. *sufS* was targeted by expressing a gene-specific sgRNA in the episomal vector pRH2521 under the control of a TetR-regulated smyc promoter (Pmyc1*tetO*). For *sufS* depletion, sgRNAs were designed for the region 52 to 75 bp of the *sufS* ORF and cloned in pRH2521. Both the plasmids pRH2502-*dCas9* and pRH2521-sgRNA were sequentially electroporated into *Mtb* and *Mtb*Δ*iscS* strains, followed by the selection of positive clones on KAN + HYG plates. The SufS^−^ KD was achieved by culturing the KD strains in 7H9 media and treating them with ATc (200 ng/ml) when OD_600_ reached 0.1 to 0.2, with the addition of ATc every 48 hours. Levels of *sufS* depletion were verified by RT-qPCR after 48 hours of ATc treatment.

### RT-qPCR analysis

Total bacterial RNA was extracted using the FastRNA Pro Blue Kit (MP Biomedicals, Santa Ana, CA) and further purified using RNeasy spin columns (Qiagen, USA) in accordance with the manufacturer’s instructions. For qRT-PCR analysis under different experiment setups, total RNA was extracted at indicated time points, followed by treatment with deoxyribonuclease (DNase). Approximately 600 ng of DNase-treated RNA was used for cDNA synthesis using random hexamer oligonucleotide primers (iScript Select cDNA Synthesis Kit, Bio-Rad, USA). Gene-specific primers and iQ SYBR Green Supermix (Bio-Rad, USA) were used for qRT-PCR (StepOne Plus, Thermo Fisher Scientific, USA). For gene expression analysis, 16*S* rRNA levels were used as internal normalization control in all cases. A list of primers used for qRT-PCR is given in table S4.

### RT-qPCR on RNA of *Mtb* derived from mouse lungs and infected macrophages

Bacterial RNA was extracted from infected mouse lungs as described in (*80*), with slight modification. Briefly, lung tissues were dissociated in a buffer containing Liberase TM (0.2 mg/ml; Roche, Basel, Switzerland), DNase I (0.1 mg/ml; Roche, Basel, Switzerland), and 5 mM MgCl_2_ for 1 hour at 37°C at 180 rpm. Cells were spun down at 5000 rpm for 15 min, and the pellet was resuspended in 1× red blood cell lysis buffer and incubated at room temperature for 10 min with intermittent mixing. Samples were centrifuged at 5000 rpm for 5 min, and the pellet was resuspended in 1 ml of RNA Pro solution (FastRNA Pro Blue Kit) to lyse most of the mammalian cells. Samples were centrifuged at 12,000*g* for 15 min at 4°C, the supernatant was carefully removed, and the pellet was resuspended again in 1 ml of RNA Pro solution and lysed by bead beating followed by chloroform-isopropanol–based isolation of RNA. The total RNA isolated was quantified, and quality was checked spectrophotometrically using NanoDrop. RNA was treated with DNase (Turbo DNase, RiboPure-Blood kit, Invitrogen), followed by first-strand cDNA synthesis using Maxima H minus RT enzyme mix (Thermo Fisher Scientific) using gene-specific primers for 16*S* rRNA and *sufS* (table S4). The cDNA obtained is amplified using Taq DNA polymerase (NEB) with gene-specific primer pairs. Last, real-time qPCR of the amplified cDNA is performed using iQ SYBR Green Supermix. For gene expression analysis, 16*S* rRNA levels were used as internal normalization control in all cases.

Bacterial RNA was isolated from infected RAW264.7 murine macrophages after 48 hours of infection as described in (*14*). Briefly, infected macrophages were harvested and treated with 5 M GTC buffer for differential lysis of only macrophages. The samples were centrifuged at 13,000 rpm for 20 min to separate the bacterial pellet. The bacterial pellet was resuspended in 1 ml of RNA Pro solution (FastRNA Pro Blue Kit) and lysed by bead beating. Total RNA isolation, cDNA synthesis, and PCR steps were followed as mentioned above. A list of primers used is given in table S4.

### Statistical analysis

All data were graphed and analyzed with Prism v9.0 (GraphPad Software, San Diego, CA) unless otherwise stated. Representative data of at least three independent biological replicates are indicated as means ± SEM. Statistical significance was determined with two-tailed unpaired *t* test or with one-way or two-way analysis of variance (ANOVA) for comparison of multiple groups. All *P* values are given in each figure legend.

### Ethics statement

This study was carried out strictly following the guidelines provided by the Committee for the Purpose of Control and Supervision on Experiments on Animals (CPCSEA), Government of India. The protocol for the animal experiment was approved by the animal ethical committee on the Ethics of Animal Experiments, Indian Institute of Science (IISc), Bangalore, India (approval number: CAF/Ethics/544/2017). All humane efforts were made to minimize the suffering.
