# Supplementary material for: Cysteine desulfurase (IscS)–mediated fine-tuning of bioenergetics and SUF expression prevents Mycobacterium tuberculosis hypervirulence
Source: Sci Adv. 2023 Dec 13;9(50):eadh2858. doi: 10.1126/sciadv.adh2858 (PMC10848736; doi:10.1126/sciadv.adh2858)
Supplement: Supplementary file 1 — Figs. S1 to S16 Legends for tables S1 to S4 [file sciadv.adh2858_sm.pdf]

Science Advances
AAAS

Supplementary Materials for

Cysteine desulfurase (IscS)–mediated fine-tuning of bioenergetics and SUF expression prevents *Mycobacterium tuberculosis* hypervirulence

Mayashree Das *et al.*

Corresponding author: Amit Singh, asingh@iisc.ac.in

Sci. Adv. **9**, eadh2858 (2023)
DOI: 10.1126/sciadv.adh2858

The PDF file includes:

Figs. S1 to S16
Legends for tables S1 to S4

Other Supplementary Material for this manuscript includes the following:

Tables S1 to S4

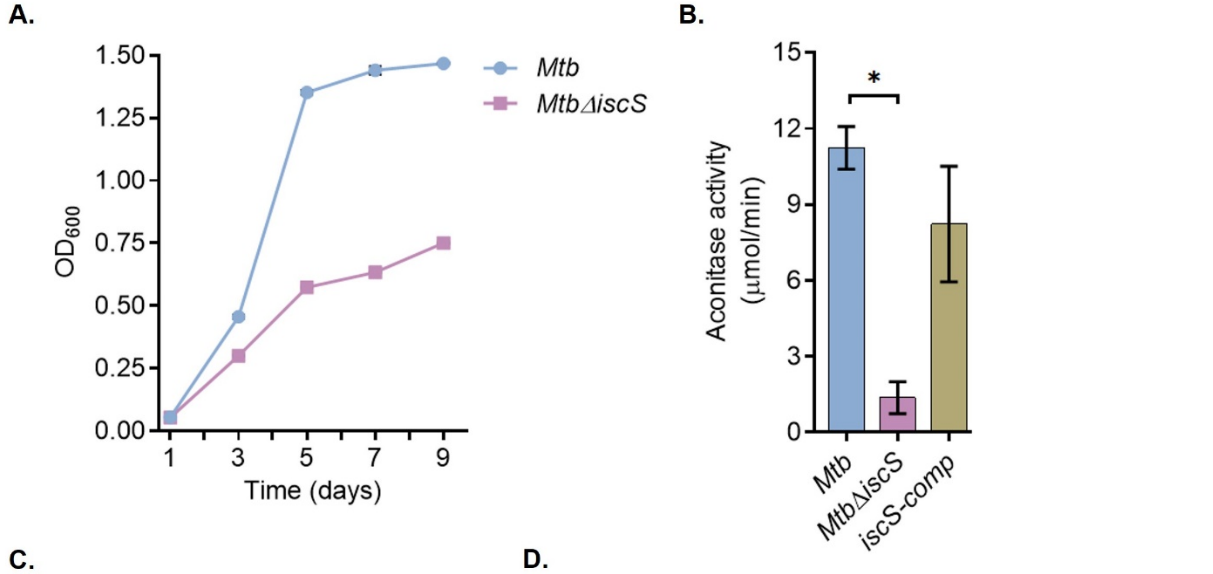

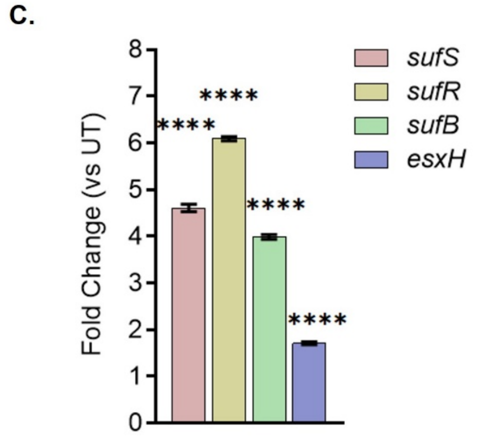

D.

Stimulus	MIC$_{90}$		
	Mtb	***MtbΔiscS***	***iscS-comp***
2,2-bipyridyl	200 µM	100 µM	200 µM
Thiourea	6.25 mM	3.125 mM	12.5 mM

Fig. S1. IscS is important for growth under aerobic conditions. (**A**) *MtbΔiscS* showed retarded growth than *Mtb* under aerobic culture conditions. (**B**) Aconitase activity was measured from cell free lysates of *Mtb*, *MtbΔiscS* and *iscS-comp* strains grown under aerobic culture conditions. (**C**) Expression of *suf* and *esxH* genes induced under iron-starvation caused by the iron- chelator, 2,2-bipyridyl by qRT-PCR. Results are expressed as mean ± standard error of mean (SEM). Data are representative of at least 2 independent experiments. (**D**) Table showing the MIC$_{90}$ values of *Mtb* strains exposed to a concentration gradient of 2,2-bipyridyl and thiourea. Results are expressed as mean ± standard error of mean (SEM). (**B**) *$p \leq 0.05$, significance determined by unpaired two-tailed *t* test. (**C**) ****$p \leq 0.0001$, significance determined by two-way ANOVA with Bonferroni's multiple comparisons test.

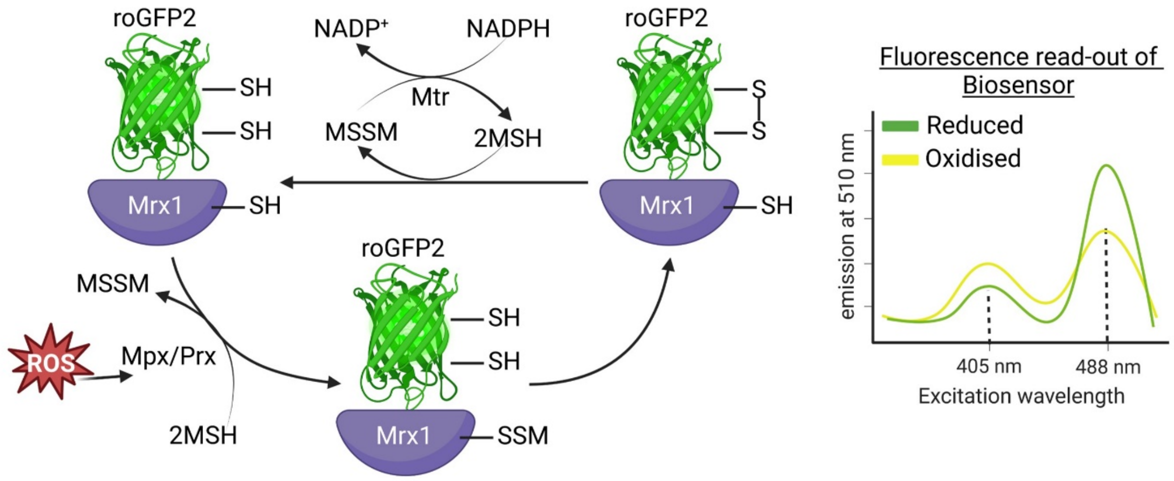

Fig. S2. Working principle of the Mrx1-roGFP2 redox biosensor. Oxidative stress skews the ratio of oxidized (MSSM) to reduced mycothiol (MSH) via a mycothiol-dependent peroxiredoxin (Prx) or peroxidase (Mpx). In response to oxidative stress, the redox active cysteine of Mrx1 reacts with MSSM to generate a mixed Mrx1-MSSM intermediate. Due to the proximity of roGFP2 to Mrx1, the Mrx1-MSSM interacts with the Cys-thiols on roGFP2 converting it to S-mycothionylated roGFP2. This intermediate rearranges to form a disulfide bond resulting in an oxidative shift in E_{MSH}. Oxidation of Mrx1-roGFP2 increases the fluorescence intensity for excitation at 405 nm and a decrease at 488 nm with a fixed emission at 510 nm. This image was created using BioRender.com.

A.

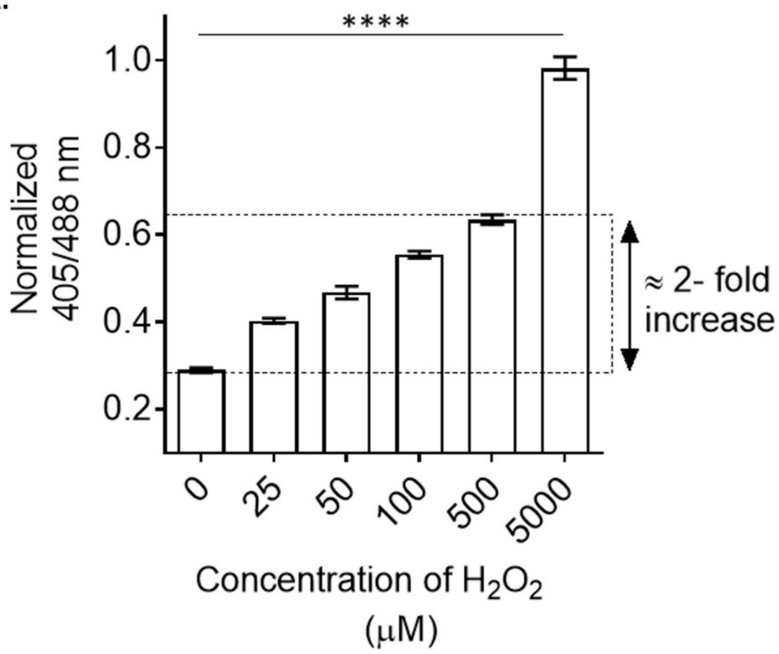

Fig. S3. Mrx1-roGFP2 measures redox changes in *M. tuberculosis* in response to H_2O_2. *M. tuberculosis* expressing Mrx1-roGFP2 was treated with the indicated concentrations of H_2O_2 for 5 min, and the ratiometric sensor response was measured by flow cytometry. A 2-fold increase in biosensor's ratiometric signal inside cells corresponds to biosensor oxidation in cells treated with 500 μM of H_2O_2 when compared to untreated cells. Error bars represent standard deviation from the mean. Data represent at least two independent experiments performed in at least duplicate. Statistical significance was analyzed over untreated control by one-way ANOVA analysis (****$p < 0.0001$) (*58*).

Copyright © 2022 American Society for Microbiology, Antimicrob. Agents Chemother.
September 2022 Volume 66 Issue 9 e00592-22, https://doi.org/10.1128/aac.00592-22

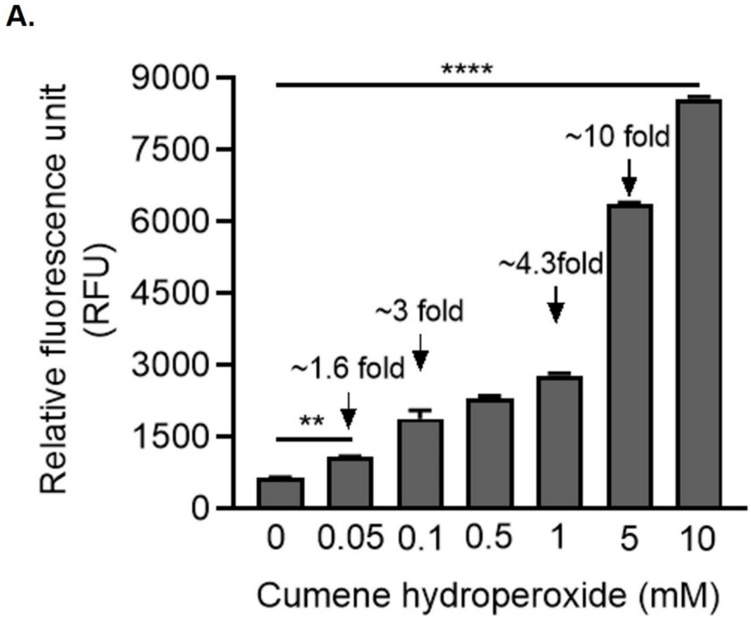

Fig. S4. Intracellular ROS measurement by CellRox in response to a concentration gradient of oxidant cumene hydroperoxide (CHP). *Mtb* treated with the indicated concentrations of CHP for 5 min, and the fluorescence response was measured by flow cytometry. A 1.5-fold increase in CellRox signal inside cells corresponds to an oxidation in cells treated with 50 μM of CHP when compared to untreated cells. Data shown are representative of two independent experiments, mean ± standard error of mean (SEM). Statistical significance was analyzed over untreated control by unpaired two-tailed *t* test (**$p < 0.01$, ****$p < 0.0001$).

A.

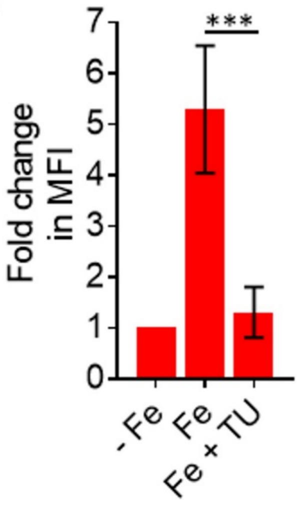

Fig. S5. Increased free iron (Fe) concentration leads to ROS generation in *M. tuberculosis*. Fe-depleted bacterial cultures (-Fe) were supplemented with 80 µM ferric chloride ($FeCl_3$) in the presence or absence of an ROS scavenger, thiourea (TU; 10 mM), for 4 days, and ROS were quantified by flow-cytometry using CellROX Deep Red dye. Data represent fold change in median fluorescence intensity (MFI) of the dye over untreated control (-Fe). Error bars represent standard deviation of the mean. Data shown are representative of two independent experiments performed in duplicate. *p* was determined by unpaired two-tailed student's t-test analyzed relative to an untreated control. (*** $p \leq 0.001$ relative to the untreated control; ns indicates not significant) (*58*).

Copyright © 2022 American Society for Microbiology, Antimicrob. Agents Chemother.
September 2022 Volume 66 Issue 9 e00592-22, https://doi.org/10.1128/aac.00592-22

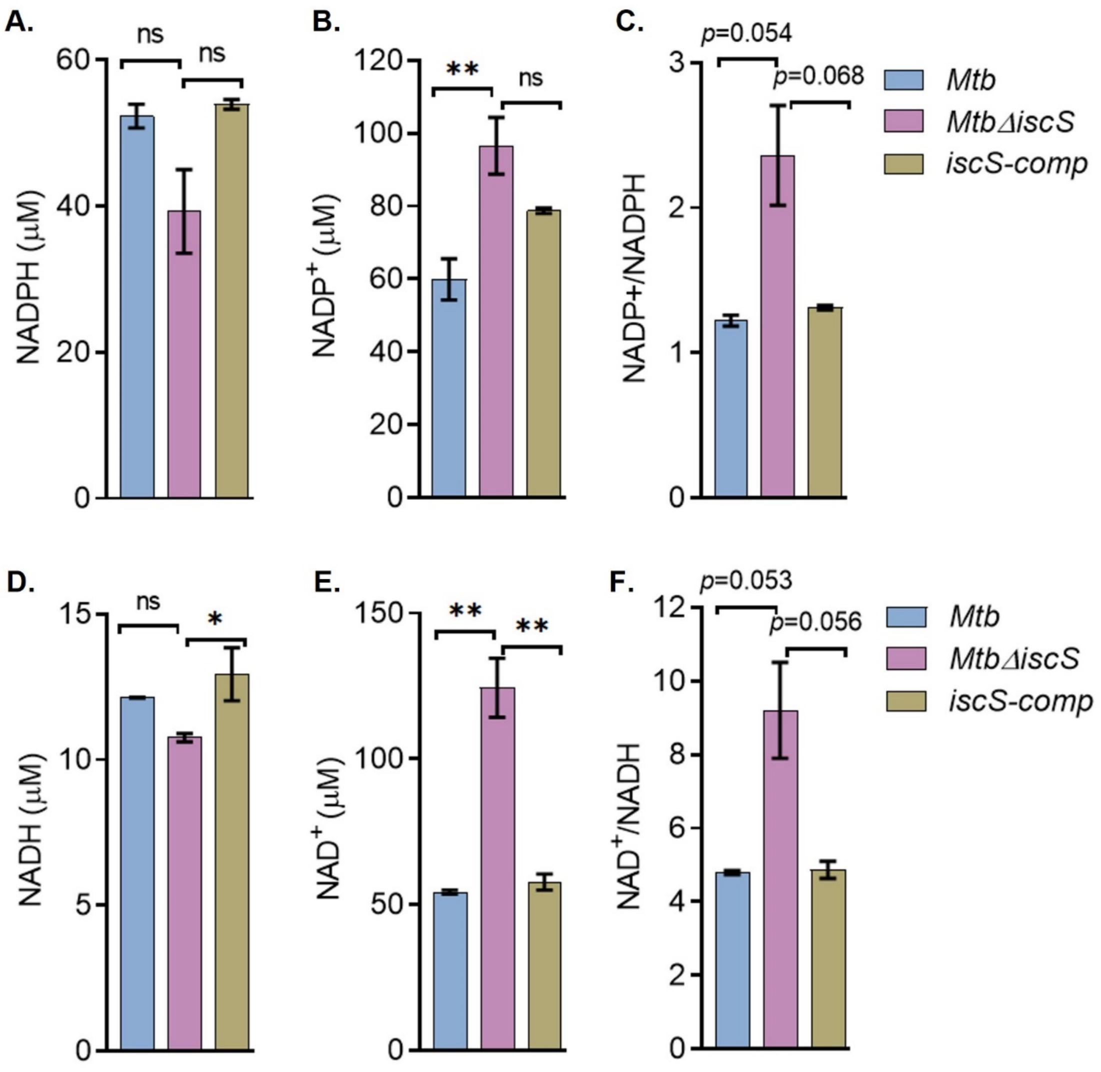

Fig. S6. Deletion of *iscS* leads to skewed ratio of cellular redox couples. Detection of NADPH (**A**), $NADP^+$ (**B**), $NADP^+$/NADPH (**C**), NADH (**D**), NAD^+ (**E**), and NAD^+/NADH (**F**) from *in vitro* grown cultures of *Mtb, MtbΔiscS*, and *iscS-comp* as described in materials and methods. Results are expressed as mean ± standard error of mean (SEM). ns: not significant, *$p \leq 0.05$, **$p \leq 0.01$ by one-way ANOVA with Bonferroni's multiple comparisons tests.

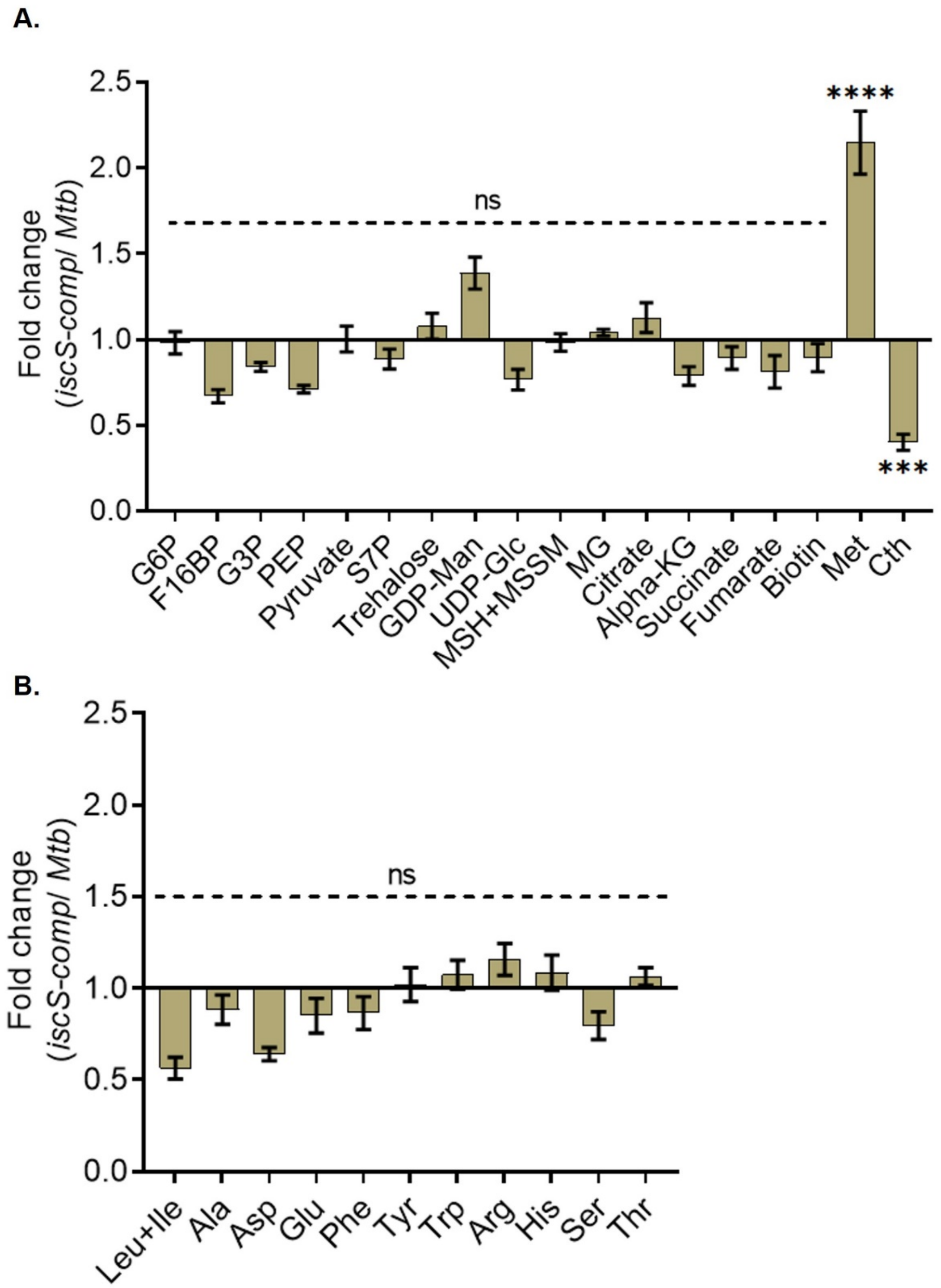

Fig. S7. Restoration of *iscS* expression in *MtbΔiscS* reverses the metabolic imbalance. LC MS/MS analysis of metabolic intermediates in the *iscS-comp* from different pathways (**A**) glycolysis, PPP, sugar nucleotides, total mycothiol, methylglyoxal, sulfur metabolites, TCA metabolites, and (**B**) amino acids. Data are represented as fold change respective to *Mtb* and mean ± standard error of mean (SEM). Experiments were performed in biological triplicate and *p* value was calculated

by two-way ANOVA with Bonferroni's multiple comparisons test compared to *Mtb* levels (ns, not significant; ***$p \leq 0.001$, ****$p \leq 0.0001$).

A.

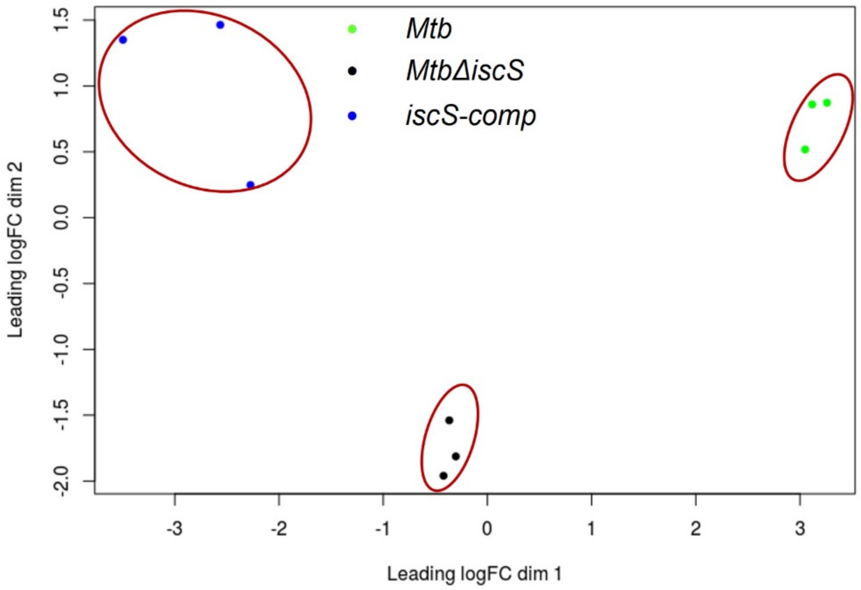

Fig. S8. Complementation of altered transcriptome of *MtbΔiscS* in IscS-Comp. (**A**) Multidimensional scaling analysis (MDS) plot shows all RNA-seq samples- *Mtb*, *MtbΔiscS*, and *iscS-comp* - clustering with their biological replicates.

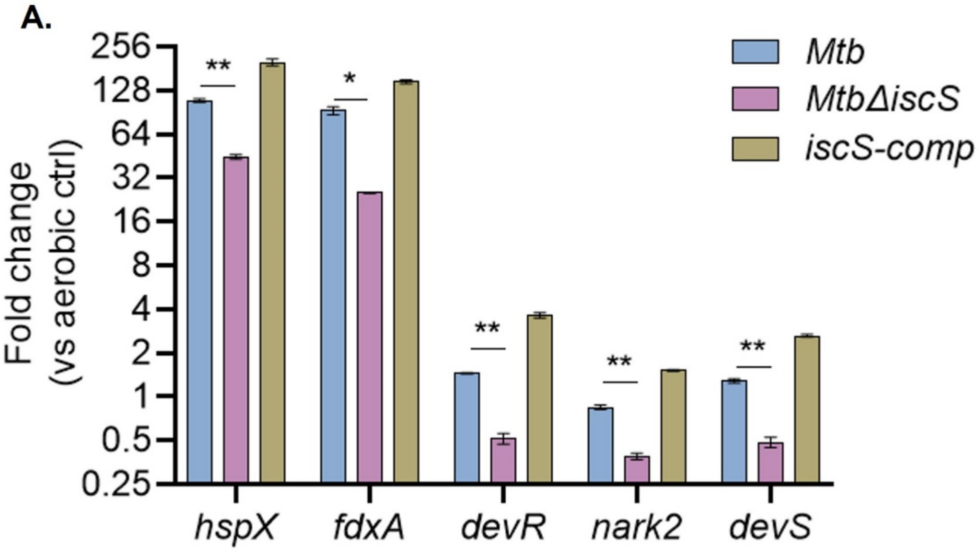

Fig. S9. Gene expression analysis using RT-qPCR of DOS-regulon genes under hypoxia. Hypoxia was achieved by culturing the mycobacterial strains with reduced head-to-space ratio following the Wayne model and RNA isolated on decoloration methylene blue (indicator of oxygen saturation). Expression levels of certain DOS-regulon genes (*hspX, fdxA, devR, narK2, devS*) were measured. **(A)** Fold change in expression compared to aerobic controls of respective strains are plotted. Results are expressed as mean ± standard error of mean (SEM). *$p \leq 0.05$, **$p \leq 0.01$, ***$p \leq 0.001$, and ****$p \leq 0.0001$ by unpaired two-tailed student's t-test.

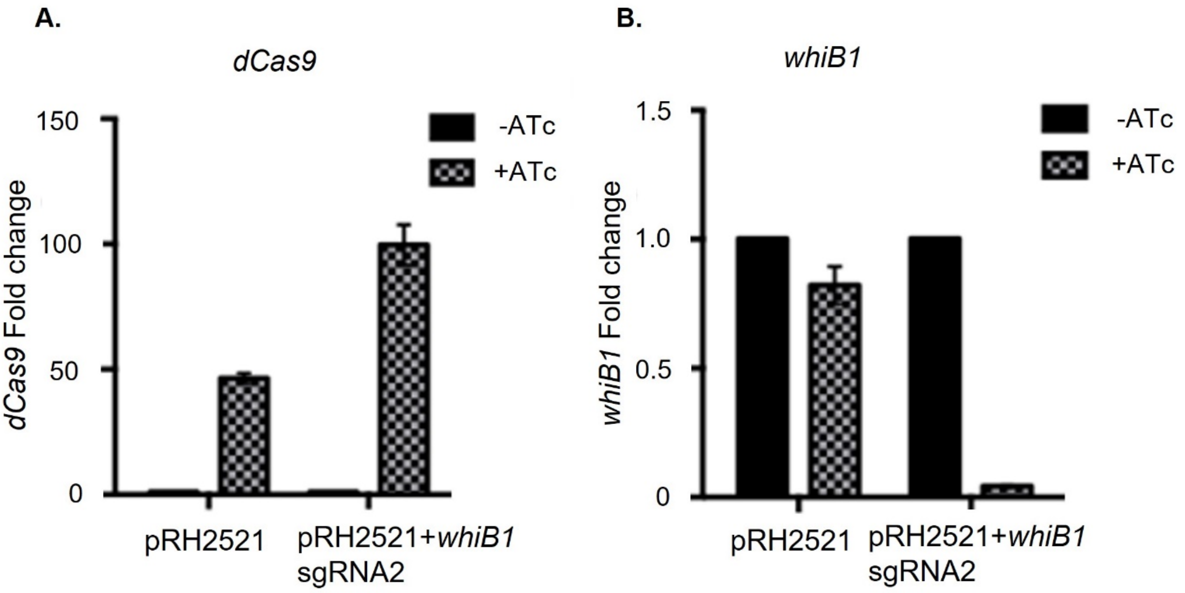

Fig. S10. Validation of WhiB1 conditional knockdown strain. Determination of gene expression of *dCas9* (**A**) and *whiB1* (**B**) in the vector control (pRH2521) and *whiB1* KD (pRH2521+whiB1 sgRNA2), with or without the exposure to anhydrotetracycline (ATc).

A.

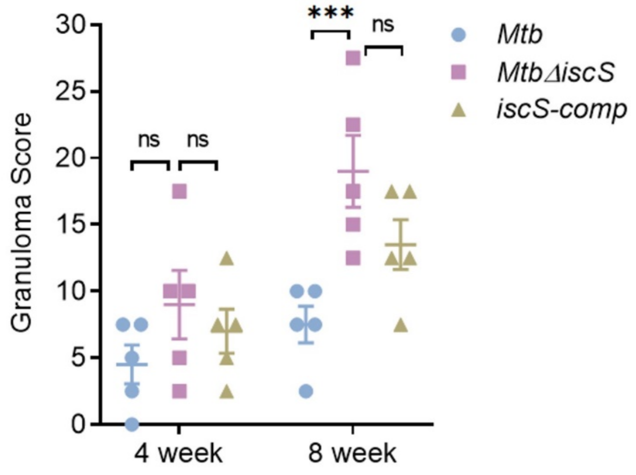

Fig. S11. Increased lung pathology in *MtbΔiscS* infected mice. (**A**) Histopathological analysis of the lung sections is shown as granuloma score observed in the infected mice-lung samples. ****p*≤0.001 by two-way ANOVA with Bonferroni's multiple comparisons test.

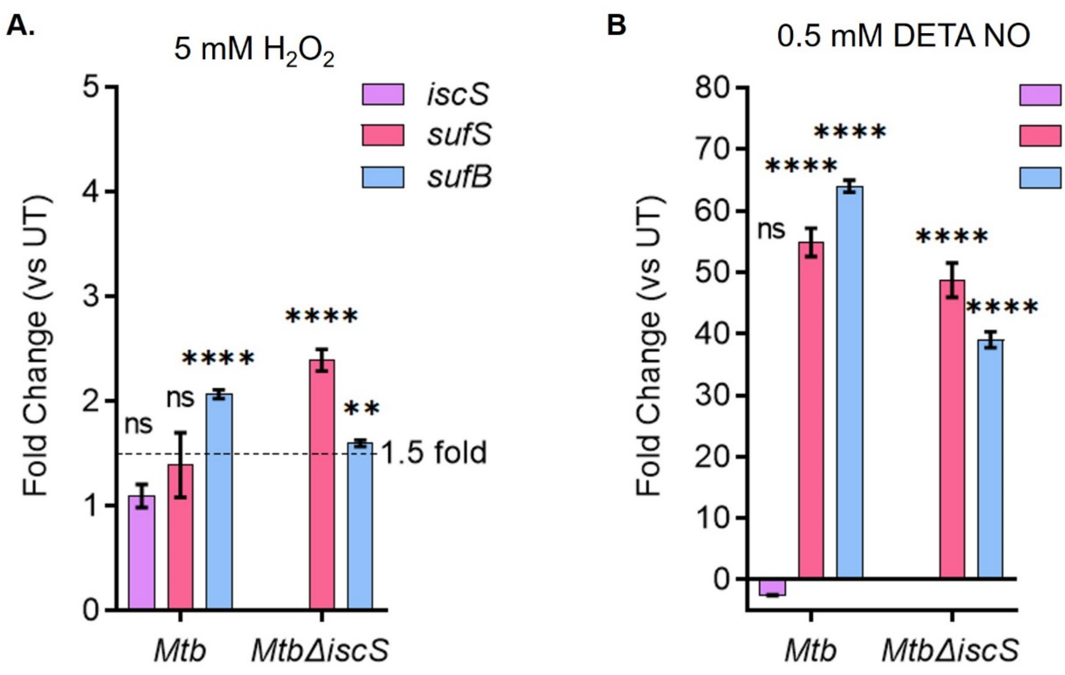

Fig. S12. Induction of *suf* genes in *Mtb* under stress conditions. (**A, B**) Wild type (WT) *Mtb* and *MtbΔiscS* cultures were exposed to **(A)** 5 mM H_2O_2 and **(B)** 0.5 mM DETA-NO for 4 h followed by total RNA isolation and expression of *sufS*, *sufB*, and iscS was determined by RT-qPCR. Fold change was measured respective to untreated samples. ***p*≤0.01 *****p*≤0.0001 by two-way ANOVA with Bonferroni's multiple comparisons test.

A.

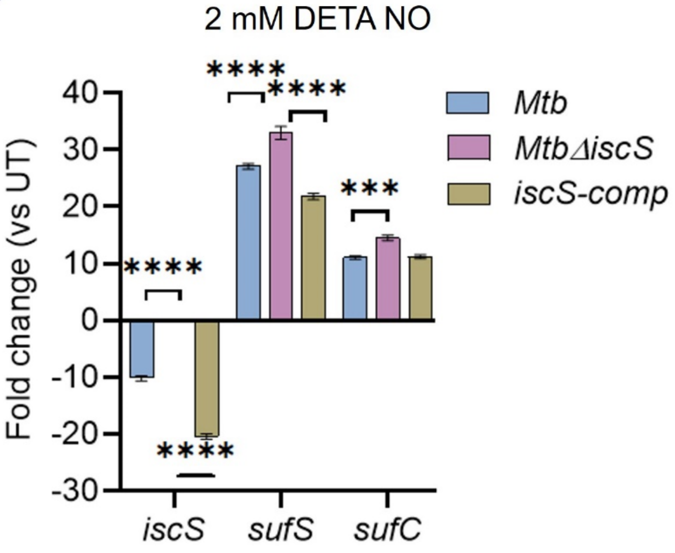

Fig. S13. Induction of *suf* genes in *Mtb* under exogenous NO stress conditions. (**A**) Wild type (WT) *Mtb* cultures were exposed to 2 mM DETA-NO for 4 h followed by total RNA isolation and expression of *sufS*, *sufB*, and *iscS* was determined by RT-qPCR. Fold change was measured respective to untreated samples. **p≤0.01 ****p≤0.0001 by two-way ANOVA with Bonferroni's multiple comparisons test.

A.

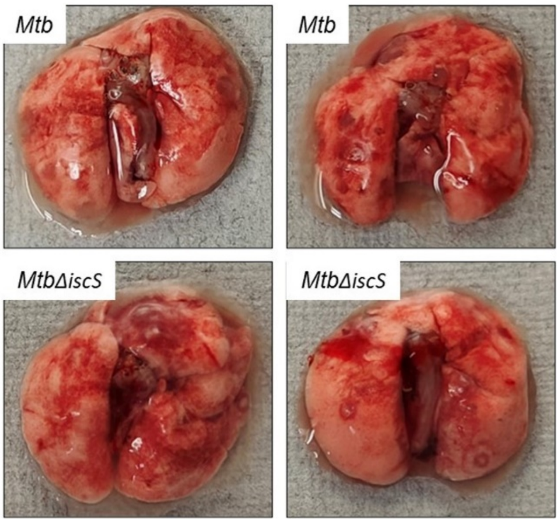

Fig. S14. The gross pathology of the infected lungs is shown post 4 weeks of infection in iNOS$^{-/-}$ mice. The gross pathology of the lungs in both *Mtb* and *MtbΔiscS* was comparable.

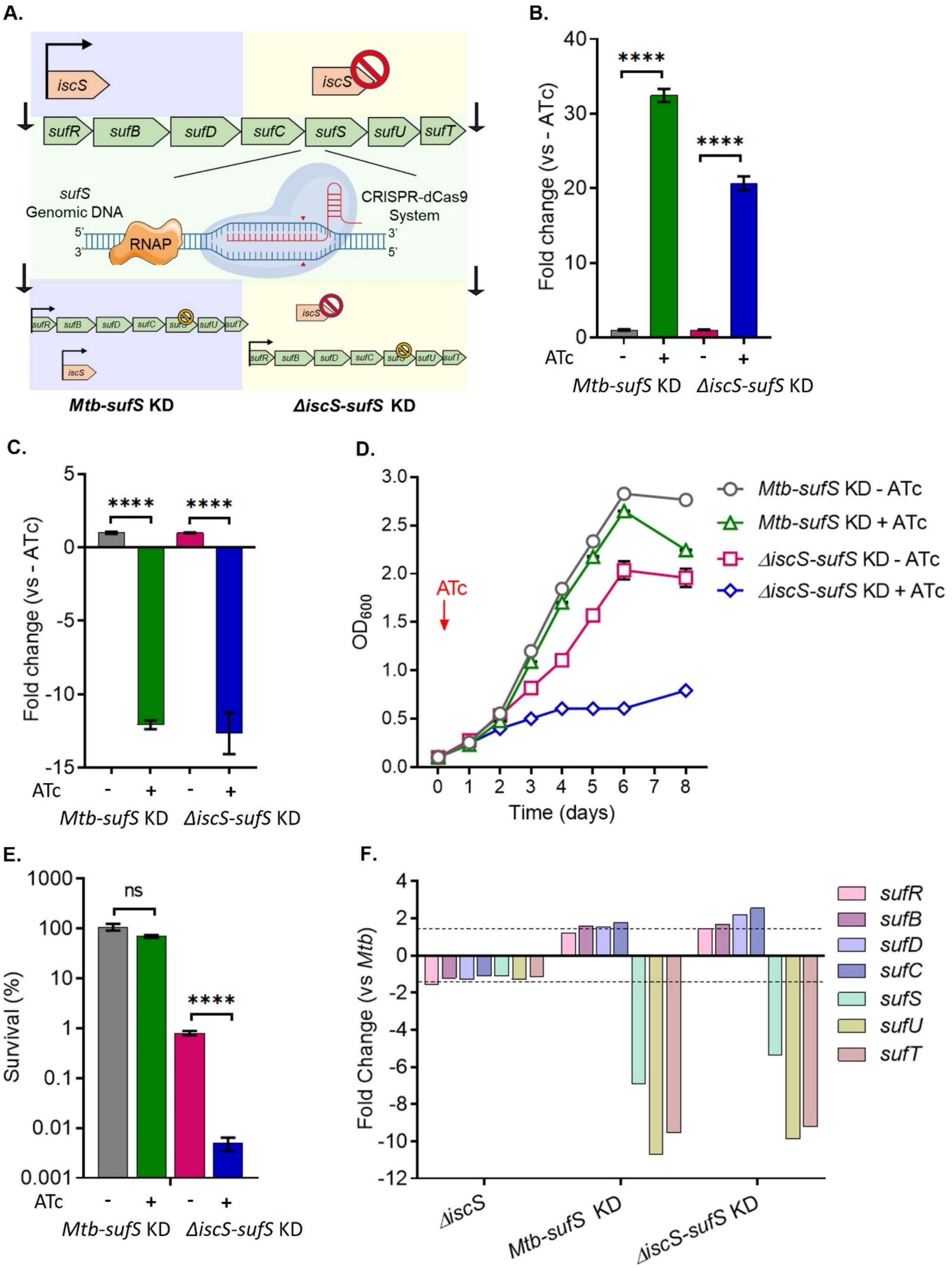

Fig. S15. Conditional knockdown of *sufS* in *MtbΔiscS*. Effect of CRISPRi-mediated silencing of the essential gene *sufS* on *in vitro* growth of *Mtb*. (**A**) A schematic for the generation of *sufS* conditional knockdown strains in wt *Mtb* and *MtbΔiscS* backgrounds using CRISPR-dCas9 mediated CRISPRi strategy. Confirmation of

dCas9 overexpression (**B**) and *sufS* downregulation (**C**) in the conditional knockdown strains on exposure to 200 ng/ml of anhydrotetracycline (ATc), determined by qRT-PCR. Data plotted as fold change relative to untreated (-ATc). (**D**) Growth was determined for *Mtb*-sufS KD (+/- ATc) and *ΔiscS-sufS* KD (+/- ATc) under aerobic condition by measuring the OD at 600 nm after treatment with 200 ng/ml Atc. (**E**) Log phase cultures of *Mtb*-sufS KD and *ΔiscS-sufS* KD (+/- ATc) were diluted to 0.2 OD and exposed to 5 mM H_2O_2 stress. Percent survival post 24 h was determined by enumerating CFU. (**F**) Expression of the *suf*- operon genes were analysed in the *MtbΔiscS*, *Mtb*-sufS KD, and *ΔiscS-sufS* KD strains by extracting RNA from exponentially growing cultures. Fold changes in expression were compared to that of *Mtb*. ****$p \leq 0.0001$ by unpaired two-tailed *t* test.

A.

Percent Lung Granuloma	Modified Score
>50%	4
11 to 50	3
4 to 10	2
1 to 3	1

B.

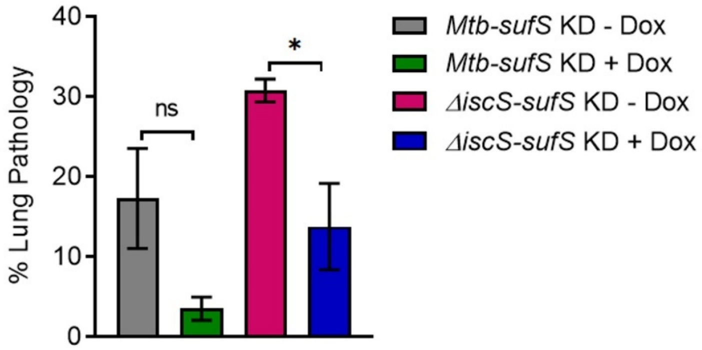

Fig. S16. Reduced lung pathology in the conditional knockdown of *sufS* in mice. (**A**) The table illustrates the modified scoring of lungs of mice infected with *Mtb* based on the severity and distribution of granuloma and infiltration of alveoli with mononuclear cells (as per reference 14 in main manuscript). (**B**) Histopathological analysis of the lung sections derived from the infected mice. Histopathological damage is quantified as percent lung pathology based on the granuloma scoring shown in panel A. *$p \leq 0.05$, ns, not significant by unpaired two-tailed *t* test.

Supplementary data files

Table S1: List of genes encoding Fe-S cluster proteins in *Mtb*

Table S2: RNA sequencing data of wt *Mtb*, *MtbΔiscS, and iscS-comp*

Table S3: The parent and daughter ion masses for each metabolites

Table S4: List of oligonucleotides used in this study
